# Police discrimination and police distrust among ethnic minority adolescents in Germany

**DOI:** 10.3389/fsoc.2024.1231774

**Published:** 2024-02-12

**Authors:** Irena Kogan, Markus Weißmann, Jörg Dollmann

**Affiliations:** ^1^University of Mannheim, School of Social Sciences, Mannheim, Germany; ^2^Mannheim Centre for European Social Research (MZES), University of Mannheim, Mannheim, Germany; ^3^GESIS-Leibniz Institute for the Social Sciences, Cologne, Germany; ^4^DeZIM-German Center for Integration and Migration Research, Berlin, Germany

**Keywords:** police discrimination, trust in police and courts, children of immigrants, Germany, survey data

## Abstract

In light of ongoing debates about racially motivated police violence, this paper examines two separate but interrelated phenomena: instances of police discrimination and mistrust in police and the judicial system among ethnic minorities in Germany. Analyses are carried out based on waves 1, 3, and 5 of the CILS4EU-DE data collected among 14 to 20 year-old respondents in Germany. The focus of the paper lies on young men from the Middle East, as well as Northern and Sub-Saharan Africa, who—as our study demonstrates—tend to disproportionally more often report discrimination experiences and particularly low levels of trust in police and courts compared to other ethnic minorities and the majority populations in Germany, and partially also in comparison to their female counterparts. We also show that more frequent experiences of police discrimination are associated with greater distrust of the police and partially also with courts among young men from the Middle East, North and Sub-Saharan Africa. Female adolescents from similar backgrounds are also more distrustful of the police, but this is not explained by their own experiences of police discrimination.

## Introduction

Following the murder of George Floyd by a Minneapolis policeman in 2020, a “Black Lives Matter” movement emerged in US and international headlines and quickly grew to become one of the largest protest movements in US history. This movement brought to the forefront numerous cases of racism, discrimination, and police violence perpetrated against African-Americans. Cases of police discrimination, police brutality, stop and search arrests, pre-trial detention directed against visible minorities, etc. have also attracted considerable research attention (Dukes and Kahn, 2017; Kahn and Davies, 2017; Buehler, 2017; Menifield et al., 2018; Edwards et al., 2019; Pierson et al., 2020). Researchers reveal heavy-handedness exhibited by the police in minority communities, highlighting a lack of respect toward African-American and other citizens of color in those communities when compared to majority-white communities (Websdale, 2001; Lundman and Kaufman, 2003). Apart from the US, there is evidence on pervasive police presence in immigrant areas, for example among Black British or other ethnic minorities in the UK (Athwal and Bourne, 2015; Vomfell and Stewart, 2021), people originating from North Africa in France or the Aboriginal people in Australia (Antonopoulos, 2003). The police stops African–American drivers more frequently than would be expected based on population averages (Walker, 2000; Lundman and Kaufman, 2003; Pierson et al., 2020), which is also supported by individuals’ self-reports (Lundman and Kaufman, 2003). Reportedly, ascriptive characteristics play a considerable role in the context of low-visibility police actions such as the decision to issue a traffic ticket following a traffic stop (Reiss, 1992; Lundman and Kaufman, 2003). Important correlates for disproportional attention of police toward ethnic and racial minorities, including the heightened use of guns by police against minorities, are shown to be young age, male gender, lower socio-economic status, and a high concentration of ethnic minorities in the neighborhood (Antonopoulos, 2003; Dukes and Kahn, 2017). In most of the research on police discrimination, male gender emerged as one of the strongest correlates (Welch, 2007; Gabbidon et al., 2011; Edwards et al., 2019).

Unsurprisingly, visible minorities and citizens of color are more inclined to be suspicious of police motives and actions during their encounters with police (Van Craen and Skogan, 2015). The overall highly visible presence of police in minority neighborhoods and the over-policing of offenses among people of color, coupled with the insufficient protection of victims of racist attacks, increases mistrust and non-acceptance of the police by the Afro-American communities in the US (Antonopoulos, 2003) and ethnic minorities in the UK (Sharp and Atherton, 2007).

Comparable research for Germany is less common, but the topic has recently received more attention in the literature (Eckert et al., 1998; Wiendieck et al., 2002; Schweer and Strasser, 2003; Behr, 2019; Abdul-Rahman et al., 2020; Müller and Wittlif, 2023). Although Germany lacks a history of slavery comparable to the US, we ask ourselves whether one should expect police discrimination and mistrust toward police and judicial system among ethnic and racial minorities in this country as well? In contrast to the UK or France, which have experienced significant migrations from the African continent in relation to their former colonial past, bulk of Germany’s ethnic minorities are the descendants of labor migrants who arrived from Turkey and North Africa between 1950 and 1970 to fill vacancies in West German industry (Kogan, 2011). Similar “guest worker” schemes were introduced by the East German socialist regime with North Vietnam, Mozambique, Angola, Cuba, North Korea and China (Bade and Oltmer, 2007). Another source of migration is of a humanitarian nature, with asylum seekers arriving in more recent years from Turkey, the African continent and Middle Eastern countries. Within the above-mentioned ethnic minority groups, there is a proportion of individuals who consider themselves and are perceived to be phenotypically different from the majority, and who―as a recent study by Müller and Wittlif (2023) shows―are more likely to report having been stopped and searched by the police in Germany.

In light of ongoing debates about racially motivated police violence, this paper aims to fill the research gap focusing on two separate but interrelated phenomena: self-reports of police discrimination and (mis)trust in police and the judicial system among ethnic minorities in Germany. We estimated gendered models following the common approach in the literature, which has found considerably gender differences in perceptions of police discrimination (e.g., Dottolo and Stewart, 2008) and trust in the police (e.g., Van Craen, 2012). Our specific focus lies on young male adolescents with origins in the Middle East, as well as Northern and Sub-Saharan Africa, who—as our study demonstrates—tend to disproportionally more often report discrimination experiences and particularly low levels of trust in police and courts compared to the majority population.

A main objective of this study is descriptive. We adopt the perspective of young adolescents and examine whether ethnic minorities, including those from the Middle East, Northern Africa, and Sub-Saharan Africa, report instances of police discrimination and harbor a sense of distrust toward police and the judicial system. We further assess whether levels of perceived discrimination and mistrust are comparable across both sexes or whether gender-specific discrimination and trust gaps among ethnic minorities exist in Germany – one of the largest European immigrant-accepting countries. Finally, we explore to what extent perceptions of police discrimination and mistrust can be explained by the purported victims’ characteristics, particularly those pertaining to the minorities’ structural, social, and cultural embeddedness into the host society.

The overall contribution of the paper is twofold. First, we focus on Germany, for which we have little evidence on whether ethnic minority and native majority youth of both genders differ in their perceptions of police discrimination and trust in the police and courts. Second, we highlight the relationship between perceptions of police discrimination and trust in the police and judicial system, and explore how it varies between native majority and minority youths of both genders.

The paper proceeds with the summary of two areas of literature. The first is related to possible explanations for the self-reported police discrimination, which is known to be a combination of a group’s exposure to actual discrimination, attributional processes, and sensibility to potential discrimination experiences (Major and O’Brien, 2005; Van Doorn et al., 2013a; Maxwell, 2015). The second strand of the literature is related to the explanations for the lower levels of trust in police exhibited by some ethnic minority groups, in particular, a possible link between police discrimination and trust in police or the judicial system (Röder and Mühlau, 2012; Van Craen, 2012; Van Craen and Skogan, 2015; Czymara and Mitchell, 2023). More generally, this literature discusses experiences of police discrimination and (mis)trust in the police and judicial system in the context of individuals’ structural, social and cultural integration in host societies and their experiences of unlawful behavior (Weitzer and Tuch, 2005; Berthelot et al., 2018). The discussion of the previous literature culminates in the formulation of the expectations to be empirically tested with the data. This is followed by a description of the data, variables and methodology. The presentation of the results is divided into three parts: descriptive evidence, multivariate results related to experiences of discrimination with the police, and young people’s trust in the police and courts. We conclude with a summary of the findings and a discussion of their implications for academic research and society at large.

### Perception of police discrimination among ethnic and racial minorities

Cases of over-policing targeted at immigrants and ethnic minorities, ethnic profiling in stop-and-search procedures, high levels of crime victimization, including “hate crimes,” as well as disparities with regard to arrests, charges, pre-trial detention, conviction, and incarceration rates between ethnic and racial minorities and the majority population, all serve as indicators of discrimination against ethnic minorities in European police and judicial systems (Antonopoulos, 2003; Holmberg and Kyvsgaard, 2003; Zauberman and Lévy, 2003; Albrecht, 2008; Bijleveld et al., 2008; Roché, 2008; FRA [European Union Agency for Fundamental Rights], 2009; Open Society Institute, 2009; Röder and Mühlau, 2012; Pierson et al., 2020; Vomfell and Stewart, 2021). Given that police discrimination is also pervasive in Germany (Jaschke, 1997; Eckert et al., 1998; Mletzko and Weins, 1999; Luff et al., 2018; Abdul-Rahman et al., 2020), the question arises whether we can capture it through ethnic minorities’ reports of discrimination experiences.

Researchers agree that reports of discrimination are likely to reveal actual instances of discrimination, but they also contain subjective evaluations of often ambiguous situations, which are not necessarily related to discrimination (Diehl et al., 2021). For example, such reports might also be related to members of ethnic and racial minority groups―particularly those facing salient ethnic boundaries―attributing negative situations to discrimination (Phinney et al., 1998; Branscombe et al., 1999) for example, as a sort of a coping strategy (Major and O’Brien, 2005) or in order to protect their self-esteem (Crocker et al., 1991). Furthermore, reports of discrimination might reflect cases of group discrimination even in the absence of individual discrimination (Lindemann, 2020). Empirically, individuals tend to perceive more group discrimination than individual discrimination, leading to overreporting of discrimination (Taylor et al., 1990).

The extent to which ethnic minority members misinterpret ambiguous situation might further depend on the level of their integration in society (Schaeffer and Kas, 2023). Portes et al. (1980) theorize that integration, in terms of acculturation, education, and language skills, enables minorities to cognitively better comprehend experiences of discrimination. Therefore, integration might increase rather than decrease perceived discrimination. Well-integrated minorities might be more strongly aware of their marginalized status (Flores, 2015), which may render them more sensitive to instances of discrimination, enabling them to better detect discriminatory behavior and more assertively demand equal treatment (Di Saint Pierre et al., 2015). Better-integrated racial and ethnic minorities are also likely to frame experiences through the lens of discrimination due to heightened expectations that stem from the society they were raised in (Silberman et al., 2007; Cooney, 2009). Exposure to media and news that report on challenges of migration and integration might also increase immigrants’ awareness of discrimination (Holtz et al., 2013; Van Doorn et al., 2013b; Karadas et al., 2017; Steinmann, 2019; Tuppat and Gerhards, 2020).

Assuming that reports of discrimination are accurate and reflect actual instances of individual discrimination, we might ask ourselves how this discrimination could be explained. The most prominent explanation for police discrimination against racial and ethnic minorities offered in the literature is statistical discrimination on part of police. A widely-used statistical discrimination approach assumes that, due to a lack of full information on individuals and in situations when quick processing of information is needed, certain group characteristics are assigned to individuals perceived to belong to the group in question (Arrow, 1972; Phelps, 1972; Aigner and Cain, 1977). In addition to the ethnicity and race, other characteristics, such as socio-economic status, prompt police discrimination in the form of social profiling (Behr, 2019). In particular, the intersectionality of several characteristics, e.g., young Afro-American men with low income, are shown to be relevant for police profiling (Dottolo and Stewart, 2008). According to police officers, statistical discrimination is a common practice and makes the police work more effective (Antonopoulos, 2003). Thus, for example, in continental Europe, the figures of pre-trial detention for foreigners are particularly high because they are expected to be more likely to flee the country before trial and, in the case of undocumented migrants, detention is the first step in the process of deportation (Albrecht, 1997). Statistical discrimination seems to be particularly frequent in high-crime minority neighborhoods due to low costs of discriminatory actions for police officers in these communities and fewer complaints on part of minorities (Weitzer, 1996; Schweer and Strasser, 2003). In districts with high immigrant shares in the German city of Duisburg, men phenotypically perceived to be foreign are more likely to be stopped and questioned (Schweer and Strasser, 2003).

Another approach―this time social-psychological―considers important underlying causes of discrimination in cognitively-based stereotypes and emotionally-charged prejudices (Fiske, 1998; Diehl and Fick, 2016). Dark skinned young men have been shown to be stereotyped as violent, criminal and dangerous (Trawalter et al., 2008; Correll et al., 2014) and associated with threat both implicitly (Payne, 2001; Maner et al., 2005) and explicitly (Cottrell and Neuberg, 2005). Threat stereotypes have been shown to be particularly strong for tall African American men (Hester and Gray, 2018). In an attempt to find explanations for this phenomenon, Trawalter et al. (2008) point to research using cognitive neuroscience methodology that has found differential activity in a brain region selectively responsive to perceived threat (Davis, 1992; Whalen, 1998) in response to men of color compared to white men (Hart et al., 2000; Wheeler and Fiske, 2005). This response is particularly pronounced in white individuals with relatively high levels of implicit racial attitudinal bias (Phelps et al., 2000; Cunningham et al., 2004).

Implicit and unconscious stereotypes are experimentally shown to be behind a so-called “shooter bias,” denoting when police officers are quicker to shoot at an Afro-American versus White person without any situational reasons for the decision (Correll et al., 2002; Dukes and Kahn, 2017; Kahn and Davies, 2017). The study by Najdowski et al. (2015) concentrates on potential victims, asking Afro-American and White participants to report how they feel when interacting with police officers. Notably, Afro-American men, but not White citizens, reported concern that police officers stereotyped them as criminals simply because of their race. Furthermore, they anticipated stereotype threat in the hypothetical police encounter and this anticipation translated into racial differences in levels of anxiety, self-regulatory efforts, and behavior that is commonly perceived as suspicious by police officers.

### Trust in police among ethnic and racial minorities

Researchers largely agree that minority citizens are more likely to be the target of disrespect, humiliation, and physical violence by the police than majority citizens (Tyler and Huo, 2002; Dukes and Kahn, 2017). Yet, due to the stronger fear of crime and higher rates of crime victimization among racial and ethnic minorities compared to the mainstream (Kennedy, 1997; Dukes and Kahn, 2017), minority residents should actually benefit from supporting the police and advocating for a sustained police presence in the neighborhoods, especially when such presence lowers crime rates. A question arises how such an intricate relationship with police on part of ethnic and racial minorities is reflected in their satisfaction with and trust toward police.

Research suggests that the degree of satisfaction with the police is lower among ethnic minorities than among the majority (white) population (Carter, 2002; Frank et al., 2005; Weitzer and Tuch, 2005). In the U.S., minorities are consistently more likely to hold negative views of police (Wortley et al., 1997; Weitzer and Tuch, 1999, 2005; Tyler and Huo, 2002; Hagan et al., 2005; Tyler, 2005; Brunson, 2007; Correia, 2010; McNeeley and Grothoff, 2016; Johnson et al., 2017; Kearns et al., 2020) and are systematically less confident than White people that police will protect them from violent crime (McCarthy, 2014). Heterogeneity among the US minorities is also acknowledged in the US research. Whereas Afro-Americans overall possess less trust and confidence in the police than White citizens (Bradford, 2011; Callanan and Rosenberger, 2011; Gabbidon et al., 2011), Hispanics have less trust and confidence in the police than White citizens (Bradford, 2011; Callanan and Rosenberger, 2011), but more than Afro-Americans (Callanan and Rosenberger, 2011). Furthermore Afro-American respondents are least trustful and confident in the courts compared to White and Hispanic respondents (Rottman and Tomkins, 1999).

Beyond the US, Heath et al. (2011) report broadly similar levels of trust in the police among native-born and first-generation immigrants, but significantly lower levels of trust among descendants of immigrants in Europe. Similar findings are reported in another European cross-national study by Röder and Mühlau (2012). These results are echoed by Pass et al.’s (2020) findings for Australia, which suggest that recently arrived immigrants have higher levels of trust in the police, while those who migrated as children and second-generation immigrants trust the police less. Piatkowska’s (2015) analyses of World Values Survey data from 50 countries reveal a slightly different result, finding slightly lower levels of trust in police among immigrants compared to the native population. Bradford et al. (2017) ascertain higher levels of trust among immigrants than among the UK-born population in England and Wales.

Some theories of trust suggest that personal experience with and knowledge of a social institution is important for the development of trust (Hardin, 2006). Others, however, emphasize that trust is developed in childhood through parental socialization and remains largely stable throughout life, being even transmitted across generations (Uslaner, 2002; Dinesen, 2013). This would suggest that experiences of police discrimination may be a key explanatory variable in understanding police mistrust among ethnic and racial minorities (Van Craen and Skogan, 2015). Those minority group members who personally experience discrimination by the police or other law enforcement agencies are less likely to trust these institutions. However, even those minority group members who have not personally experienced police discrimination may distrust the police or judicial system because they have been socialized (at least in part) within a community that used to or continues to experience police discrimination. As a result, they may have witnessed discrimination against other community members or even strangers with whom they share a skin color or ethnic background. Alternatively, they may be confronted with discrimination indirectly, through hearing the testimony of alleged victims from their circle of friends or family (Deutsches Zentrum für Integrations- und Migrationsforschung (DeZIM), 2022, 30 f.). According to Tyler (2006), discriminatory treatment is perceived by minorities as a failure to value them as equal members of society; it violates norms of impartiality and may also be associated with a lack of respectful and dignified treatment.

Other correlates for perceptions of and trust toward police are demographic factors, such as class, time since arrival and neighborhood characteristics (Weitzer, 1999; Weitzer, 2000; Wu et al., 2009; Taylor et al., 2015; Bradford et al., 2017). Gender differences in trust have not been emphasized thus far (but see Van Craen, 2012). Whereas neighborhood safety and crime are shown to be responsible for explaining the differences between Latino and White citizens in the US, they cannot fully explain dissatisfaction with police on part of Afro-Americans. Trust in police was shown to be higher in neighborhoods with higher proportion of immigrants in England and Wales (Bradford et al., 2017). In the study by Weitzer and Tuch (2005), frequent exposure to media coverage of incidents of police misconduct reduces individual satisfaction with police. In Lawrence’s (2000) study, the effect was, however, significant only among African Americans, which could be explained by the fact that the majority of media reports on police misconduct involved mistreatment of Afro-American rather than White or Hispanic people. Socialization experiences abroad are shown to predict trust in the host country among first-generation immigrants: Menjívar and Bejarano’s (2004) explanation to the ethnic groups’ differences in trust toward police is related to the variation in negative experiences with repressive authorities in the immigrants’ countries of origin. Czymara and Mitchell (2023) further argue that police size (relative to the population size) in immigrants’ countries of origin is negatively associated with their trust in police.

### Research questions and hypotheses

Pursuing explorative goals, the paper first seeks to detect the minority-majority gaps in perceptions of police discrimination and trust in police and courts in Germany. Second, it examines the extent to which there are minority/majority differences in these outcomes, controlling for observable differences in the characteristics of the groups. Based on the theoretical approaches addressing causes of discriminatory behavior and hitherto findings on gendered patterns of police discrimination among ethnic and racial minorities, we expect visible minorities, particularly those originating from MENA and sub-Saharan countries (*Hyp. Discrimination 1*) and particularly men (*Hyp. Discrimination 2*), to report more frequent police discrimination. It remains to be explored to what extent these gaps are reduced when controlling for indicators of their structural, social, and cultural integration into German society, as well as instances of unlawful behavior.

We also hypothesize that visible minorities, particularly those from MENA and sub-Saharan countries (*Hyp. Distrust 1*), will be more distrustful of the police and justice system. We expect minorities’ experiences of discrimination to explain (at least in part) their distrust of the police and courts (*Hyp. Distrust 2*).

### Data and methods

The following analyses are based on data from waves 1 and 3 of the German part of the Children of Immigrants Longitudinal Survey in Four European Countries (CILS4EU) and the fifth wave of its German extension (CILS4EU-DE) (Kalter et al., 2016, 2019, 2021). The first three waves were conducted within an international research framework, including England, the Netherlands, and Sweden as the other participating countries. From wave 4 onwards, country studies were conducted independently of each other on the basis of the initial country samples. The sample was selected following a school-based probability sampling approach, with the sampling units being on three levels: schools, classes, and students. First, in the school year 2010/11, a sample of schools was selected from a list comprising all schools of a country enrolling the relevant target population, i.e., students enrolled in a school class, in which most of the students were already, or would turn, 14 during the school year. In order to achieve a sufficient number of students with an immigrant background in the final sample, schools with a higher proportion of immigrants were oversampled. Furthermore, implicit stratifies were used to achieve proportionate samples across school types and regions. Within the selected schools, two classes were randomly selected, and within the selected classes, all students were asked to participate in the survey. This strategy resulted in the selection of 144 schools, 271 school classes, and 5,013 students in the first wave in Germany.

Three dependent variables are in focus of the analyses. The first one pertains to the individuals’ feeling of being discriminated against by police/security guards, which is measured on a four-point scale ranging between “never” to “always”. All respondents who participated in wave 1 and/or wave 3 (unbalanced sample) are included into the analyses. Since our other outcome variables are collected in wave 5, we restrict our sample to those who participated in wave 5 and answered at least one of the two trust questions in wave 5.^1^

The second dependent variable is trust in police, whereas the third dependent variable is trust in courts, both measured in wave 5 on a four-point scale with the extreme categories “not at all strongly” and “very strongly.” For the analyses of trust in law-enforcement and judicial institutions, we restricted the sample to those who participated in the survey in wave 3 and wave 5.

The analyses are carried out by means of the OLS regression with robust standard errors. The choice of the OLS regression is explained by the ease with which focal coefficients can be compared across nested models. Additionally, we cross-checked the results with the help of the ordinal logistic regression (results of the ordinal logistic regressions are found in the Supplementary Appendix and discussed in comparison with OLS regression results).

The following regression equation illustrates our analytical approach and includes the set of key independent variables discussed in more detail below:


$$\begin{array}{l} y=\beta_{0}+\beta_{1}\;W-EUR/USA+\beta_{2}FSU/CEE+\beta_{3}\mathrm{MENA}\\ \;+/\mathrm{Africa}+\beta_{4}\;\mathrm{Other}+\beta_{5}\mathrm{Male}\;+\beta_{6}W-EUR/USA\\ \;\times \mathrm{Male}+\beta_{7}FSU/CEE\times \mathrm{Male}+\beta_{8}\mathrm{MENA}+/\mathrm{Africa}\\ \;\times \mathrm{Male}+\beta_{9}\;\mathrm{Other}\times \mathrm{Male}+\zeta +\epsilon \text{.} \end{array}$$

The key independent variables are respondents’ ethnic origin and gender. We distinguish between native majority youth (a benchmark category) and immigrants or children of immigrants arriving from: (a) Northern, Western, and Southern Europe as well as the US (abbreviated *W-EUR/USA* in the equation above), (b) countries of the Former Soviet Union and Eastern Europe (including countries formerly constituting the Yugoslavian Republic) (abbreviated *FSU/CEE*), (c) Turkey, Middle Eastern (including Afghanistan and Pakistan), and African countries, which include both North Africa and Sub-Saharan Africa (abbreviated *MENA+/Africa*), (d) other origins.

Gender is measured in a dichotomous way, with women as the reference group. In addition to the main effects of ethnic origin and gender, we include a set of interaction terms between the two. In this way, the (conditional) main effects of ethnic origin refer to the average female respondent. The gender effect (abbreviated as *Male* in the equation above) represents a mean difference between majority native-born men and women in the respective outcome variable. The interaction terms between ethnic origin groups and gender (e.g., *W- EUR/USA* × *Male*) refer to mean differences between male respondents of the respective origin groups and the majority of native-born males.

The modeling is organized stepwise. In Model 1 we include only focal independent variable ethnic origin and gender and the interactions between the two. In Model 2, in addition to the focal independent variables we control for a set of background characteristics (marked by *ζ* in the equation above). These are, first, demographic characteristics, such as adolescents’ generational status. We distinguish between respondents who migrated themselves versus all others. Additional demographic characteristics included in the analyses are immigrants’ age at migration^2^ and respondents’ year of birth (before 1995, during 1995, after 1995).^3^

Second, in order to assess the endowment of familial resources – for instance, social networks, cultural capital, and economic resources – we control for families’ socio-economic characteristics. These include the parents’ highest level of education, differentiating between no degree, secondary, and tertiary levels of education (reference category). In addition, we include a dummy variable to capture individuals without information about their parents’ education. Parental occupational status is represented by the highest ISEI (International Socio-Economic Index of Occupational Status) (Ganzeboom et al., 1992) score of both parents.^4^ This information was taken from wave 1.

Third, we account for languages spoken at home in order to establish the extent of cultural resources in the family. We complement this with the measure of TV consumption in a language other than German. In addition to the measurement of cultural capital, this item also captures the extent of individual orientation with the heritage country.^5^ Finally, we capture individual religious background differentiating between the group of individuals with no religious denomination (reference category), Christianity, Islam, and other religions (Hinduism, Judaism, Buddhism, Yezidism). Answers to the question “How important is religion to you”, with four answer categories ranging from 0 (religion is not at all important) to 3 (religion is very important) reflect the degree of religious affinity. Both religious affiliation and religiosity reflect a general set of values and attitudes, which in turn may be related to perceptions of discrimination and trust.

Fourth, we further take into account social capital resources available outside the family, such as share of native-born friends and share of natives in the neighborhood (as the latter information was administered only in wave 1, we used this information also for wave 3). Both are captured by a five-point-scale ranging from “almost all or all” to “none or very few”. The extent of social embeddedness outside the family may shape individual perceptions and attitudes, including those related to trust in social institutions.

Obviously, encounters with the police might be explained by young people’s unlawful activities, such as deliberate damage to property, steeling, carrying a knife or a weapon, or being very drunk. This is accounted for in Model 3. We additionally control for frequency of drinking, smoking, and drug usage.^6^ Once controlling for unlawful activities and accounting for the individuals’ background characteristics, we compare perceptions of police discrimination among otherwise comparable individuals and hence come closer to detecting actual discrimination.

In the fourth model of the analyses pertaining to *trust in police or the judicial system*, we control for police discrimination, for which we take the maximum value per person in either wave 1 or 3. The aim of such analyses is to establish to what extent police discrimination is responsible for minorities’ distrust in police or the court system.

All independent variables in the models of the police discrimination analyses are constructed on the basis of the information collected in Wave 1 and Wave 3 (unless otherwise stated), except for time-constant information (such as gender, year of birth or age at migration, and ethnic origin). In the models of the analyses relating to trust in the police or the justice system, time-varying information was taken from Wave 3 (with the exception of and experience of police discrimination, see above), which is used as an independent variable in the models predicting trust in the police or the courts.

In all models we take account of the particularities of the sampling procedure. This is done by controlling for the following characteristics of the sampled school in wave 1: school type (lower secondary school, intermediate secondary school, multi-track school, comprehensive school, upper secondary school, special needs school, Rudolf Steiner school), the proportion of immigrants in the school (0–10%, 10–30%, 30–60%, 60–100%), and the federal state of the school.

### Descriptive findings

**Figure 1:**
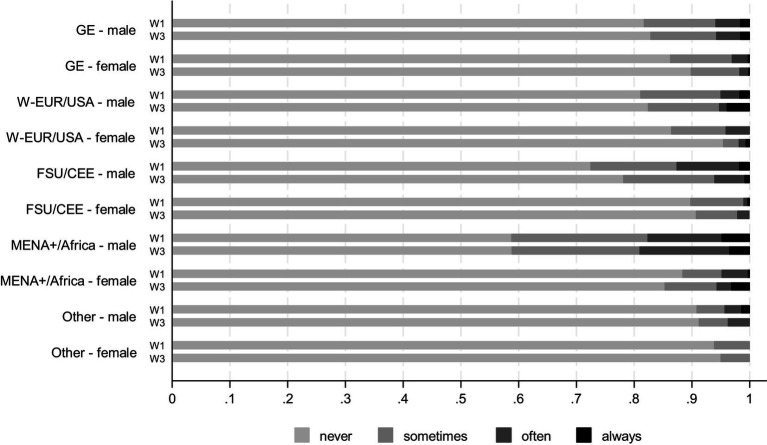
Unfair treatment by police and security guards by origin groups and gender, distribution of answers. CILS4EU, waves 1–3 (1.2.0, 2.3.0, 3.3.0), unbalanced sample, weighted results. W1 = Wave 1, W3 = Wave 3.

We start with the distribution of answers to the question about experiences of unfair treatment by policy and security guards. These are depicted in Figure 1 based on wave 1 and 3 of the CILS4EU data separately by gender groups (Figure A1 plots the same distribution without gender differentiation). The figure demonstrates that the majority of teenagers never experienced any discrimination by police or security guards. Yet, there is a considerable variation across the groups with regard to rare, frequent, and enduring experience of discrimination.

Of all the groups, more male teenagers of MENA+ and African origin report discrimination, particularly frequent and constant discrimination. Somewhat lower shares of young men with origins in FSU and Eastern Europe report police discrimination, and these shares are somewhat higher compared to the rest of young people. Male teenagers are considerably more likely to report discrimination across all analyzed groups. The gender gaps are particularly large among teenagers originating from Eastern European, MENA+, and African countries. In contrast, there are smaller differences between majority young men and women in reported discrimination experiences.

Whereas more teenagers report discrimination around ages 14 to 15 (in wave 1), the prevalence of such reports decreases as they reach ages 16–17 (in wave 3). A single exception are teenagers of MENA+ and African origin; for these groups, the share of those reporting discrimination remains consistently high (among young men) or even increase (among young women).

**Figure 2:**
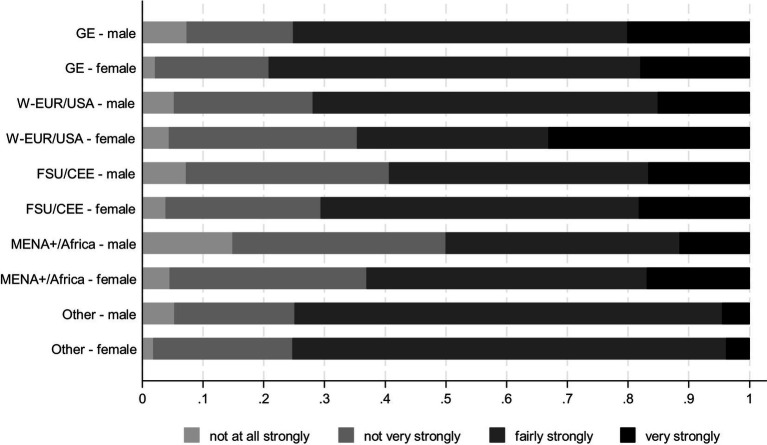
Trust in police by origin groups and gender, distribution of answers. CILS4EU, Wave 5, wave 3 sample, weighted results.

**Figure 3:**
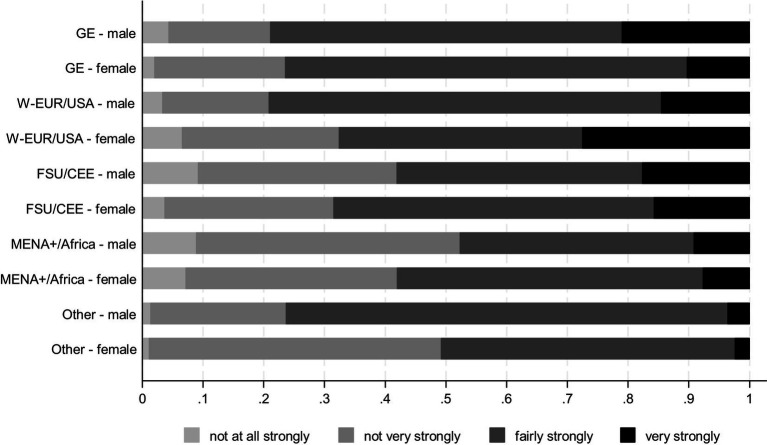
Trust in courts by origin groups and gender, distribution of answers. CILS4EU, Wave 5, Wave 3 sample, weighted results.

Whether young men and women differ in their trust in police and courts can be seen in Figures 2, 3 respectively (Figures A2, 3 plot distributions without gender differentiation). Whereas the differences in patterns of trust in police across male and female respondents who constitute the majority native-born are not that pronounced, the patterns of trust in police for young people with a migration background tend to be stronger gendered. Large shares of female teenagers with origins in Western Europe and the US tend to both distrust police and have a very strong trust in police, much unlike their male counterparts. In contrast, male teenagers from CEE, MENA+, and African countries distrust police more strongly than female respondents do.

The gendered patterns of trust in courts mimic those reported for trust in police with a single major exception in the group of minorities from other origins. Unlike trust in police, trust in courts seems to be considerably higher among male teenagers compared to female teenagers in this group of respondents.

### Is there indication of police discrimination against male visible minority teenagers?

In the next step, we run multivariate OLS regression models with individuals’ feeling of being discriminated against by police/security guards serving as a dependent variable. Table 1 contains results of this modeling, which is constructed in the following way. Model 1 encompasses coefficients which capture the gaps in perceived discrimination between female respondents of various origin groups and the native-born (see main effects of origin groups). It further contains coefficients pertaining to the gender gap in perceived police discrimination among the native-born majority (main effect of gender) as well as the gaps between male respondents in each origin group and the native-born majority men (interaction effects of gender). In Model 2, all these gaps are shown net of the individuals’ socio-demographic characteristics, social, and cultural resources. In Model 3, they are estimated also net of the instances of deviant behavior. A full set of coefficients can be found in Table A1 in the Appendix, whereas selected results of an alternative specification—an ordinal logistic regression—are reported in Table A2 in the Appendix. The deviations between OLS regression results and those from the ordinal logistic regressions are discussed below.

**Table 1:** Selected coefficients from OLS random effects regression with the dependent variable Feeling discriminated against by police/security guards.

	Model 1: gross effect	Model 2: + background characteristics	Model 3: + unlawful behavior
*Origin groups (ref: native)*			
W-EUR/USA	−0.05	−0.02	−0.01
	(0.04)	(0.05)	(0.05)
x male	0.05	0.04	−0.02
	(0.08)	(0.08)	(0.07)
FSU/CEE	−0.04	−0.06	−0.02
	(0.03)	(0.04)	(0.04)
x male	0.14^*^	0.14^*^	0.10^+^
	(0.06)	(0.06)	(0.06)
MENA+/Africa	−0.07^*^	−0.13^*^	−0.09^+^
	(0.03)	(0.06)	(0.05)
x male	0.34^***^	0.34^***^	0.29^***^
	(0.06)	(0.06)	(0.06)
Other	−0.08^*^	−0.10^*^	−0.06
	(0.04)	(0.05)	(0.05)
x male	0.15	0.19^+^	0.15
	(0.11)	(0.11)	(0.10)
Male	0.08^**^	0.07^**^	0.03
	(0.02)	(0.02)	(0.02)

CILS4EU, waves 1–3 (1.2.0, 2.3.0, 3.3.0), unweighted results. Unbalanced sample of respondents in wave 1 and wave 3 with information on either trust in police or trust in courts in wave 5.

+ *p* < 0.1, * *p* < 0.05, ** *p* < 0.01, *** *p* < 0.001; panel-robust standard errors in parentheses. *N* = 4,185. Control variables encompass immigrant proportion in school in wave 1 (W1), school type in wave 1 (W1) (Model 1), year of birth, migrant generation, age at migration, religious denomination, importance of religion, parents’ highest education, parents’ highest ISEI, TV consumption in foreign language, share of native-born in neighborhood, share of native-born friends (Model 2), deliberately damaged others’ things, stolen something from shop/other people, carried a knife or weapon, how often drink alcohol, how often smoke cigarettes, how often use drugs (Model 3), feeling discriminated in school, feeling discriminated in train/busses, feeling discriminated in shops/stores (Model 4). Full set of coefficients can be found in Table A1 in the Appendix.

Overall, our results indicate that female teenagers with migration roots do not differ much from the majority native-born girls and young women. Only female respondents of MENA+ origin and from other countries are less likely to report discrimination by police and the estimated gaps are statistically significant at either 5% or 10% levels—depending on whether OLS or the ordinal logistic regression is applied—before and after controlling for the background characteristics (Model 2, Table 1). Once we control for instances of unlawful behavior, the lower propensity of MENA+/African girls to report police discrimination (compared to the benchmark of majority native-born girls) becomes weakly significant in OLS models and disappears altogether in ordinal logistic regression models (see Table A2 in the Appendix). These results allow us to conclude that the lower incidence of unlawful behavior among MENA+/African girls could explain their lower levels of reporting police discrimination compared to the majority native-born girls. Finally, a negative effect for the group of “other” girls in Model 1 and Model 2 turns out to be insignificant (also in the ordinal logistic regression, see Table A2 in the Appendix) and is therefore not discussed.

The majority native-born male teenagers are more likely to report discrimination than the majority native-born female teens, which is visible from the main gender effect (labeled as “male” in Table 1). Teenage boys of MENA+/African origin are significantly more likely to report police discrimination, and the gap to the native-born German male teenagers is not reduced once controlling for the background characteristics (in Model 2). Teenage boys with MENA+/African heritage are still significantly more likely to report discrimination once instances of unlawful behavior are controlled for (Model 3). This means that at comparable levels of (reported) unlawful behavior, male MENA+/African minorities are more likely to experience police discrimination. Our results further suggest that FSU/CEE minorities are also more likely to report discrimination. However, these significant differences compared to the native-born disappear when the frequency of unlawful behavior is taken into account in the ordinal logistic regression model and remain significant only at the 10% level in the OLS analyses. Otherwise, the above-described results are comparable to those found in the ordinal logistic regression models.

For robustness, we also compared the results of the ordinal logistic models with those estimated for the sample of Waves 1–3, i.e., with less attrition (see Table A3 in the Appendix). The results for girls are largely robust, whereas for male teenagers we observe persistence of positive effects among the FSU/CEE group in all estimated models. Also for the group of “other” male teenagers, the positive coefficients are statistically significant in Models 2–3 and statistically significant at the 10% level in Model 1.

Our results demonstrate that young people who mention deliberately damaging things, carrying a knife, smoking frequently or consuming alcohol or drugs are more likely to report police discrimination (see Table A1 in the Appendix). This means that engaging in unlawful behavior or frequently abusing substances is associated with reports of more frequent discrimination by police officers.

Finally, we looked at potential heterogeneity within the MENA+/African group and the results are found in Table A4 in the Appendix. These results show that the gaps in reported discrimination are similar between MENA+ and Turkish young men. However, while the higher rates of reported discrimination persist for MENA+ regardless of the control variables included, there is a substantial reduction in the gap for Turkish teenage boys. We do not find significant positive gaps with native teenage males for boys from sub-Saharan Africa, which may be due to the small sample size for this group.

### Do minorities distrust police and courts?

In the next step, we pursue the question of whether, in light of possible police discrimination, minority youth distrust police and courts more than the majority native-born teenagers. To this end, we run OLS regression models with trust in police (Table 2) and trust in courts (Table 3) as dependent variables. The models in Tables 2, 3 are built similarly to those found in Table 1. Additionally, we ran alternative model specifications, i.e., ordinal logistic regressions (see Table A5 for trust in police and Table A8 for trust in courts in the Appendix), results of which we mention if we observe considerable discrepancies to the OLS models.

**Table 2:** Selected coefficients from OLS regressions with the dependent variable trust in police.

	Model 1: gross effect	Model 2: + background characteristics	Model 3: + unlawful behavior	Model 4: + experience of police discrimination
*Origin groups (ref: native)*				
W-EUR/USA	−0.06	−0.04	−0.05	−0.07
	(0.10)	(0.10)	(0.10)	(0.10)
x male	−0.12	−0.06	−0.01	−0.01
	(0.14)	(0.14)	(0.14)	(0.14)
FSU/CEE	−0.09	−0.05	−0.07	−0.09
	(0.07)	(0.08)	(0.08)	(0.08)
x male	0.02	−0.02	0.01	0.05
	(0.11)	(0.11)	(0.11)	(0.10)
MENA+/Africa	−0.16^**^	−0.13	−0.16^+^	−0.19^*^
	(0.06)	(0.10)	(0.09)	(0.09)
x male	−0.18^+^	−0.22^*^	−0.18^+^	−0.07
	(0.10)	(0.09)	(0.10)	(0.09)
Other	−0.25^+^	−0.24^+^	−0.25^+^	−0.27^*^
	(0.13)	(0.14)	(0.14)	(0.13)
x male	0.15	−0.01	0.01	0.05
	(0.21)	(0.20)	(0.20)	(0.20)
Male	−0.10^*^	−0.09^*^	−0.06	−0.05
	(0.04)	(0.04)	(0.04)	(0.04)

CILS4EU, waves 1–3 (1.2.0, 2.3.0, 3.3.0), unweighted results, wave 3 sample.

+ *p* < 0.1, * *p* < 0.05, ** *p* < 0.01, *** *p* < 0.001; robust standard errors in parentheses. *N* = 2,386. Control variables encompass immigrant proportion in school in wave 1 (W1), school type in wave 1 (W1) (Model 1), year of birth, migrant generation, age at migration, religious denomination, importance of religion, parents’ highest education, parents’ highest ISEI, TV consumption in foreign language, share of native-born in neighborhood, share of native-born friends (Model 2), deliberately damaged others’ things, stolen something from shop/other people, carried a knife or weapon, how often drink alcohol, how often smoke cigarettes, how often use drugs (Model 3), feeling discriminated against by police/security guards (Model 4). Full set of coefficients can be found in Table A7 in the Appendix.

**Table 3:** Selected coefficients from OLS regressions with the dependent variable trust in courts.

	Model 1: gross effect	Model 2: + background characteristics	Model 3: + unlawful behavior	Model 4: + experience of police discrimination
*Origin groups (ref: native)*				
W-EUR/USA	0.01	0.06	0.04	0.03
	(0.09)	(0.10)	(0.10)	(0.10)
x male	−0.13	−0.10	−0.06	−0.06
	(0.13)	(0.13)	(0.13)	(0.13)
FSU/CEE	−0.01	0.07	0.05	0.05
	(0.06)	(0.08)	(0.08)	(0.08)
x male	−0.18^+^	−0.21^*^	−0.18^+^	−0.16
	(0.10)	(0.10)	(0.10)	(0.10)
MENA+/Africa	−0.12^+^	−0.02	−0.03	−0.05
	(0.06)	(0.09)	(0.09)	(0.09)
x male	−0.19^*^	−0.23^*^	−0.20^*^	−0.14
	(0.09)	(0.09)	(0.09)	(0.09)
Other	−0.21^+^	−0.18	−0.18	−0.19
	(0.12)	(0.12)	(0.12)	(0.12)
x male	0.12	0.00	0.02	0.04
	(0.18)	(0.17)	(0.17)	(0.17)
Male	0.09^*^	0.10^**^	0.11^**^	0.12^**^
	(0.04)	(0.04)	(0.04)	(0.04)

CILS4EU, waves 1–3 (1.2.0, 2.3.0, 3.3.0), unweighted results, wave 3 sample.

+ *p* < 0.1, * *p* < 0.05, ** *p* < 0.01, *** *p* < 0.001; robust standard errors in parentheses. *N* = 2,363. Control variables encompass immigrant proportion in school in wave 1 (W1), school type in wave 1 (W1) (Model 1), year of birth, migrant generation, age at migration, religious denomination, importance of religion, parents’ highest education, parents’ highest ISEI, TV consumption in foreign language, share of native-born in neighborhood, share of native-born friends (Model 2), deliberately damaged others’ things, stolen something from shop/other people, carried a knife or weapon, how often drink alcohol, how often smoke cigarettes, how often use drugs (Model 3), feeling discriminated against by police/security guards (Model 4). Full set of coefficients can be found in Table A10 in the Appendix.

With regard to trust in police, Model 1 suggests somewhat lower levels of trust among native-born young boys compared to girls. A discrepancy between the statistically significant coefficients in the OLS regression (Table 2) versus marginally significant coefficients the ordinal logistic regression (Table A5) is however notable. Furthermore, female teenagers with origins in MENA+, African, and other countries have lower trust in police than their native-born counterparts, albeit the effect for the “other” minority group is statistically significant at the 10% level (see Model 1, Table 2). No significant differences between young minority and majority men are noticeable in Model 1, i.e., without controlling for respondents’ background characteristics. The only exception is a group of MENA+/African origin young men, who appear to have slightly lower levels of trust than the majority of male teenagers, although the coefficient is significant at the 10% level. Background characteristics partially explain the effect for girls of MENA+/African origin, so that they are no longer significantly different from the majority native girls (see Model 2, Table 2). The gap in mistrust of the police among MENA+/African origin teenage boys widens and becomes statistically significant once background characteristics are taken into account. Controlling for instances of unlawful behavior (see Model 3, Table 2) explains a large part of the gender gap among the majority of natives. Part of the explanation for the particularly low levels of trust in the police among MENA+ young men is also related to their unlawful behavior, but the effects remain pronounced for this group even after controlling for this variable, being however statistically significant only at the 10% level. A tendency to mistrust the police, which can be observed among teenage girls from MENA+/African backgrounds, becomes more pronounced when instances of unlawful behavior are taken into account, and the coefficient becomes marginally significant. Finally, when we control for experiences of discrimination in model 4, the negative coefficient for MENA+ and African male respondents disappears, suggesting that for this group a somewhat higher level of mistrust of the police is related to negative experiences with the police. At the same time, there remains a gap in trust in the police between MENA+/African girls and the benchmark of German-born girls. In ordinal logistic regressions, the coefficients are not significant in Model 3 and marginally significant in Model 4 in Table A5 in the Appendix. There are also some minor discrepancies for the “other” group, which is, however, too small and heterogeneous to be meaningfully interpreted. Otherwise, the results reported above are robust to an alternative specification—the ordinal logistic regression, the results of which can be found in Table A5 in the Appendix.

Overall and in contrast to male respondents, distrust in police for female adolescents from MENA+, African, and other countries cannot be explained by the same set of factors as applied in explaining the male gaps in police trust. After controlling for experiences of police discrimination, the negative effect for female MENA+/African youth remains significant or significant at the 10% level, as in the ordinal logistic regression.

It is important to note that MENA+/African young people are far from being a homogeneous group. Results from Table A6 in the Appendix show that among young girls, those from sub-Saharan Africa are the most distrustful of the police. At the same time, male descendants of Turkish immigrants are the most distrustful of the police among young men.

In terms of important covariates of trust in police, the following results are worth mentioning (see Table A7 in the Appendix). Individuals, who have fewer native-born friends or reside in neighborhoods with none or very few majority native-born residents, are more likely to distrust police. Those who migrated to Germany later in life tend to trust police more strongly. Young people who consume drugs, as well as frequent smokers, are more distrustful of police. Finally and not surprisingly, more frequent reports of police discrimination are associated with stronger distrust in police.

Turning to the analyses of the adolescents’ trust in courts (see Table 3), we observe an overall larger trust in the judicial system among the majority native-born young men compared to women. This effect persists regardless of the control variables included in the model. We further observe that female adolescents of MENA+ and African origins are overall more distrustful of courts (with the effect being statistically significant at the 10% level in the OLS regression and 5% in the ordinal logistic regression found in Table A8), but this effect is explained by the adolescents’ background characteristics. Noteworthy is a relatively strong distrust of courts among adolescent men with origins in Eastern European, MENA+, and African countries, with the effect for the former being statistically significant at the 10% level. Overall, this effect is hardly reduced after including the background characteristics (Model 2) and instances of unlawful behavior (Model 3). Including experiences of police discrimination (Model 4) renders the effect insignificant for adolescent men from MENA+ and African countries^;^ yet the respective coefficient remains significant at the 10%-level in the ordinal logistic regression analysis, albeit being reduced in size (see Table A8). Including covariates does little to the estimate for adolescent men from Eastern European countries, which remains comparable in size and level of statistical significance (10% level) throughout models 1 through 4 in the ordinal logistic regression. In the OLS regression model, the estimate for adolescent men from Eastern European countries is no longer statistically significant in the final model (see Table 3).

Further results indicate that the key driver behind the effect of young men with background in MENA+/African countries is a stronger distrust toward judicial system among male descendant of Turkish immigrants (see Table A9).

Finally, similar to the analyses of police distrust, young people residing in neighborhoods with larger shares of majority native-born residents and those with more native-born friends are more trustful of courts (see Table A10 in the Appendix). Young people who frequently smoke cigarettes or mention deliberately damaging things, i.e., are engaged in (partially) unlawful behavior are more distrustful of judicial system. Lastly, more frequent reports of police discrimination are associated with stronger distrust in courts.

## Summary and discussion

In light of ongoing debates about racially motivated police violence in Western countries, this paper examines two separate but—as our study shows—interrelated phenomena: instances of police discrimination and mistrust in police and courts among ethnic minorities in Germany. Our specific focus lies on young men from the Middle East, Northern and Sub-Saharan Africa (MENA+/Africa), who—as our study demonstrates—tend to disproportionally more often report discrimination experiences and particularly low levels of trust in police and the judicial system compared to other ethnic minorities and the majority population in Germany.

Based on the representative CILS4EU-DE data (waves 1, 3, and 5) collected among 14 to 20 years-old respondents in Germany, we demonstrate that male teenagers with origins in Middle East and Africa are more likely to report police discrimination. These effects persist even after considering background characteristics and instances of unlawful behavior. No comparable effects are found for MENA+ teenage girls.

Furthermore, we find that descendant of immigrants from MENA+ and African countries—regardless of gender—distrust police significantly more than comparable adolescents from families without migration background. Once controlling for experiences of discrimination, the negative coefficient for male respondents from MENA+ and African countries is reduced considerably and is no longer statistically significant, suggesting police discrimination being an important part of the explanatory mechanism for police mistrust. For this group, a somewhat higher distrust in police is associated with personal negative experiences with police. In contrast to male respondents, distrust in police for female adolescents from MENA+, African, and to some extent also other countries cannot be explained by the same set of factors as applied in explaining the male gaps in police trust.

Noteworthy is a relatively strong distrust of courts among adolescent men with origins in Eastern European, MENA+, and African countries, which can also be fully or almost fully (as in ordinal logistic regressions) accounted for by their experiences police discrimination. Female descendants of the immigrant groups considered in this study do not express significantly different levels of mistrust in the German legal system compared to the majority of native girls.

Taken together, our results thus accord with the Discrimination Hypothesis 2 and Distrust Hypotheses 1 and 2. Discrimination Hypothesis 1 is not supported by our findings, as female adolescents with origins in MENA+ countries tend to report any discrimination even less frequently than the majority native-born girls. In sum, our findings are rather conclusive regarding more salient perceptions of police discrimination among young men with origins in Middle Eastern and African countries and their stronger distrust in police and courts. The extent to which statistical discrimination or other forms of discrimination by police officers or security guards play a role in young men’s experiences of discrimination cannot be answered with the data available and remains an open research question. Distrust in the law enforcement and judicial systems among these individuals is, to a large extent, related to their experiences of police discrimination and accords with Hardin’s (2006) theoretical ideas. At a social level, it is alarming that experiences of discrimination among young male teenagers at least partially explain a more general mistrust of the police and judicial system several years later. Another alarming finding is that female descendants of immigrants from a wide range of countries, who largely do not face police discrimination, still trust the police much less than their native-born counterparts. The extent to which this effect can be traced back to family socialization (Uslaner, 2002), indirect experiences of discrimination (Deutsches Zentrum für Integrations- und Migrationsforschung (DeZIM), 2022), reflects experiences from abroad (Menjívar and Bejarano, 2004; Czymara and Mitschell, 2023) or collective experiences of police discrimination among people of color awaits future investigation.

Our focus was on the perspective of young adolescents in their formative years (14 to 20 years old). Although teenagers are presumably not likely to have many violent encounters with police, their impressions of and experiences with police, as well as their trust in police and the judicial system, might leave long-lasting imprints and affect their integration in the host society. Moreover, even with the focus on young respondents, we confirm shared experiences of police discrimination and patterns of trust among young men of color, particularly those from Turkey and MENA countries, echoing findings in US research on African American and Hispanic men. Apparently, negative experiences of people of color with the police are pervasive and potentially trigger mistrust in this and other institutions.

By focusing on the perspective of young adolescents on police discrimination and trust in police and the judicial system, we obviously overlook the other side—the perspective of the police itself. This approach sidesteps a considerable body of literature on racism within the police (for the German research see: Eckert et al., 1998; Mletzko and Weins, 1999; Wiendieck et al., 2002; Schweer and Strasser, 2003; Behr, 2019; Kemme et al., 2020). Linking both the perspectives of minorities and the police would undoubtedly be a fruitful direction for future research, as it is likely to provide more conclusive evidence on the extent and importance of police discrimination in ethnic minorities’ lives.

## Data availability statement

The datasets presented in this study can be found in online repositories. The names of the repository/repositories and accession number(s) can be found at: https://doi.org/10.4232/cils4eu.5353.3.3.0, https://doi.org/10.4232/cils4eu-de.6655.6.0.0.

## Ethics statement

The CILS4EU-study obtained ethical approval from the ethical vetting boards of the Universities of Stockholm and Oxford. University of Mannheim followed the same ethical standards as the other research teams. Furthermore, informed consent prior to participating in the survey was provided by all study participants.

## Author contributions

All authors listed have made a substantial, direct, and intellectual contribution to the work and approved it for publication.

## Footnotes

^1^We carry out additional analyses in the appendix, in which we do not restrict the sample to participants in Wave 5. The comparison of both regression analyses enables us to assess the extent to which panel attrition affects the results of our analyses.

^2^This variable was set to ‘0’ for natives and German-born children of immigrants (i.e., second generation migrants).

^3^Information on gender and year of birth was taken from Waves 1, 2 and 3. In the case of conflicting information, mode information was used. When mode information was missing due to item non-response or temporary dropout, these cases were excluded.

^4^For parents’ highest education and highest ISEI, we include the highest observed value of the father and the mother. If information is available on only one parent, this information was used as proxy. This information was taken from the youth interviews in wave 1. If no information was available from respondents, we used information from parent interviews in wave 1 if available.

^5^This combined variable was based on Wave 1 information on respondents’ daily TV viewing on a 5-point scale ranging from "no time at all" to "more than 2 h a day". In Wave 1 and Wave 3, respondents were also asked whether they spoke a language other than German at home. If they reported such a language and also reported watching TV in this other language "often" or "always", they were assigned to their TV viewing response from wave 1. If they reported watching TV in this other language only "sometimes" or "never", they were coded as watching foreign TV "no time at all". Finally, respondents who reported speaking no other language at either Wave 1 or Wave 3 were assigned to a separate category "no other language than German spoken at home".

^6^These questions were not asked in one federal state in wave 1.
