# Supplementary material for: Police discrimination and police distrust among ethnic minority adolescents in Germany
Source: Front Sociol. 2024 Feb 12;9:1231774. doi: 10.3389/fsoc.2024.1231774 (PMC10898247; doi:10.3389/fsoc.2024.1231774)
Supplement: Supplementary file 1 [file Data_Sheet_1.pdf]

**Tables and figures**

Table 1: Selected coefficients from OLS random effects regression with the dependent variable Feeling discriminated against by police/security guards

| | Model 1: gross effect | Model 2: + background characteristics | Model 3: + unlawful behaviour |
|---|---|---|---|
| Origin groups (ref: native) | | | |
| W-EUR/USA | -0.05 | -0.02 | -0.01 |
| | (0.04) | (0.05) | (0.05) |
| x male | 0.05 | 0.04 | -0.02 |
| | (0.08) | (0.08) | (0.07) |
| FSU/CEE | -0.04 | -0.06 | -0.02 |
| | (0.03) | (0.04) | (0.04) |
| x male | 0.14* | 0.14* | 0.10+ |
| | (0.06) | (0.06) | (0.06) |
| MENA+/Africa | -0.07* | -0.13* | -0.09+ |
| | (0.03) | (0.06) | (0.05) |
| x male | 0.34*** | 0.34*** | 0.29*** |
| | (0.06) | (0.06) | (0.06) |
| Other | -0.08* | -0.10* | -0.06 |
| | (0.04) | (0.05) | (0.05) |
| x male | 0.15 | 0.19+ | 0.15 |
| | (0.11) | (0.11) | (0.10) |
| Male | 0.08** | 0.07** | 0.03 |
| | (0.02) | (0.02) | (0.02) |

Source: CILS4EU, waves 1-3 (1.2.0, 2.3.0, 3.3.0), unweighted results. Unbalanced sample of respondents in wave 1 and wave 3 with information on either trust in police or trust in courts in wave 5.

Note: + $p < 0.1$, * $p < 0.05$, ** $p < 0.01$, *** $p < 0.001$; panel-robust standard errors in parentheses. N=4,185. Control variables encompass immigrant proportion in school in wave 1 (W1), school type in wave 1 (W1) (Model 1), year of birth, migrant generation, age at migration, religious denomination, importance of religion, parents' highest education, parents' highest ISEI, TV consumption in foreign language, share of native-born in neighborhood, share of native-born friends (Model 2), deliberately damaged others' things, stolen something from shop/other people, carried a knife or weapon, how often drink alcohol, how often smoke cigarettes, how often use drugs (Model 3), feeling discriminated in school, feeling discriminated in train/buses, feeling discriminated in shops/stores (Model 4). Full set of coefficients can be found in Table A1 in appendix.

Table 2: Selected coefficients from OLS regressions with the dependent variable trust in police

| | Model 1: gross effect | Model 2: + background characteristics | Model 3: + unlawful behaviour | Model 4: + experience of police discrimination |
|---|---|---|---|---|
| Origin groups (ref: native) | | | | |
| W-EUR/USA | -0.06 | -0.04 | -0.05 | -0.07 |
| | (0.10) | (0.10) | (0.10) | (0.10) |
| x male | -0.12 | -0.06 | -0.01 | -0.01 |
| | (0.14) | (0.14) | (0.14) | (0.14) |
| FSU/CEE | -0.09 | -0.05 | -0.07 | -0.09 |
| | (0.07) | (0.08) | (0.08) | (0.08) |
| x male | 0.02 | -0.02 | 0.01 | 0.05 |
| | (0.11) | (0.11) | (0.11) | (0.10) |
| MENA+/Africa | -0.16** | -0.13 | -0.16+ | -0.19* |
| | (0.06) | (0.10) | (0.09) | (0.09) |
| x male | -0.18+ | -0.22* | -0.18+ | -0.07 |
| | (0.10) | (0.09) | (0.10) | (0.09) |
| Other | -0.25+ | -0.24+ | -0.25+ | -0.27* |
| | (0.13) | (0.14) | (0.14) | (0.13) |
| x male | 0.15 | -0.01 | 0.01 | 0.05 |
| | (0.21) | (0.20) | (0.20) | (0.20) |
| Male | -0.10* | -0.09* | -0.06 | -0.05 |
| | (0.04) | (0.04) | (0.04) | (0.04) |

Source: CILS4EU, waves 1-3 (1.2.0, 2.3.0, 3.3.0), unweighted results, wave 3 sample.

Note: + p < 0.1, * p < 0.05, ** p < 0.01, *** p < 0.001; robust standard errors in parentheses. N=2,386. Control variables encompass immigrant proportion in school in wave 1 (W1), school type in wave 1 (W1) (Model 1), year of birth, migrant generation, age at migration, religious denomination, importance of religion, parents' highest education, parents' highest ISEI, TV consumption in foreign language, share of native-born in neighborhood, share of native-born friends (Model 2), deliberately damaged others' things, stolen something from shop/other people, carried a knife or weapon, how often drink alcohol, how often smoke cigarettes, how often use drugs (Model 3), feeling discriminated against by police/security guards (Model 4). Full set of coefficients can be found in Table A7 in appendix.

Table 3: Selected coefficients from OLS regressions with the dependent variable trust in courts

| | Model 1: gross effect | Model 2: + background characteristics | Model 3: + unlawful behaviour | Model 4: + experience of police discrimination |
|---|---|---|---|---|
| Origin groups (ref: native) | | | | |
| W-EUR/USA | 0.01 | 0.06 | 0.04 | 0.03 |
| | (0.09) | (0.10) | (0.10) | (0.10) |
| x male | -0.13 | -0.10 | -0.06 | -0.06 |
| | (0.13) | (0.13) | (0.13) | (0.13) |
| FSU/CEE | -0.01 | 0.07 | 0.05 | 0.05 |
| | (0.06) | (0.08) | (0.08) | (0.08) |
| x male | -0.18+ | -0.21* | -0.18+ | -0.16 |
| | (0.10) | (0.10) | (0.10) | (0.10) |
| MENA+/Africa | -0.12+ | -0.02 | -0.03 | -0.05 |
| | (0.06) | (0.09) | (0.09) | (0.09) |
| x male | -0.19* | -0.23* | -0.20* | -0.14 |
| | (0.09) | (0.09) | (0.09) | (0.09) |
| Other | -0.21+ | -0.18 | -0.18 | -0.19 |
| | (0.12) | (0.12) | (0.12) | (0.12) |
| x male | 0.12 | 0.00 | 0.02 | 0.04 |
| | (0.18) | (0.17) | (0.17) | (0.17) |
| Male | 0.09* | 0.10** | 0.11** | 0.12** |
| | (0.04) | (0.04) | (0.04) | (0.04) |

Source: CILS4EU, waves 1-3 (1.2.0, 2.3.0, 3.3.0), unweighted results, wave 3 sample.

Note: + p < 0.1, * p < 0.05, ** p < 0.01, *** p < 0.001; robust standard errors in parentheses. N=2,363. Control variables encompass immigrant proportion in school in wave 1 (W1), school type in wave 1 (W1) (Model 1), year of birth, migrant generation, age at migration, religious denomination, importance of religion, parents' highest education, parents' highest ISEI, TV consumption in foreign language, share of native-born in neighborhood, share of native-born friends (Model 2), deliberately damaged others' things, stolen something from shop/other people, carried a knife or weapon, how often drink alcohol, how often smoke cigarettes, how often use drugs (Model 3), feeling discriminated against by police/security guards (Model 4). Full set of coefficients can be found in Table A10 in appendix.

Figure 1: Unfair treatment by police and security guards by origin groups and gender, distribution of answers

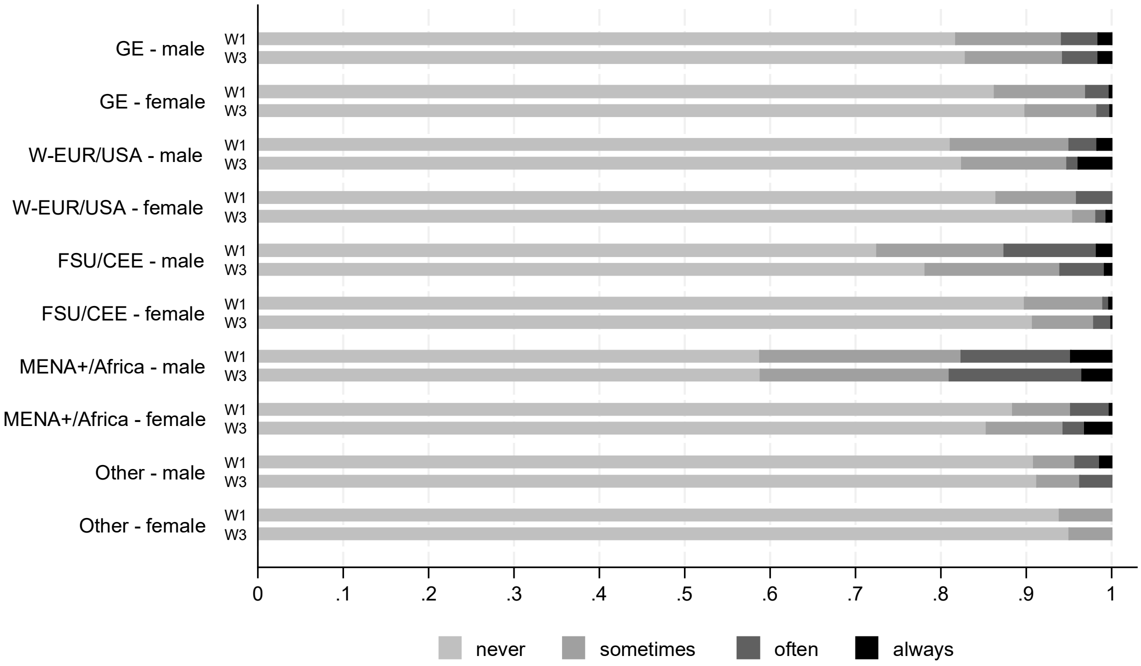


Source: CILS4EU, waves 1-3 (1.2.0, 2.3.0, 3.3.0), unbalanced sample, weighted results.
Notes: W1=Wave 1, W3=Wave 3.

Figure 2: Trust in police by origin groups and gender, distribution of answers

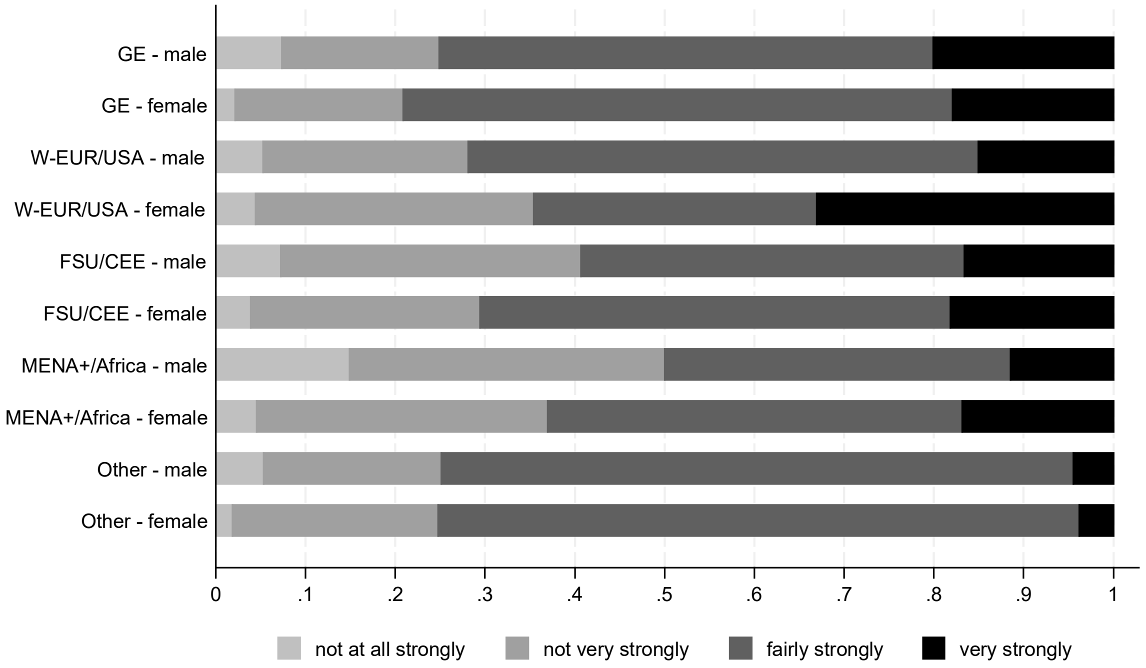


Source: CILS4EU, wave 5, wave 3 sample, weighted results.

Figure 3: Trust in courts by origin groups and gender, distribution of answers

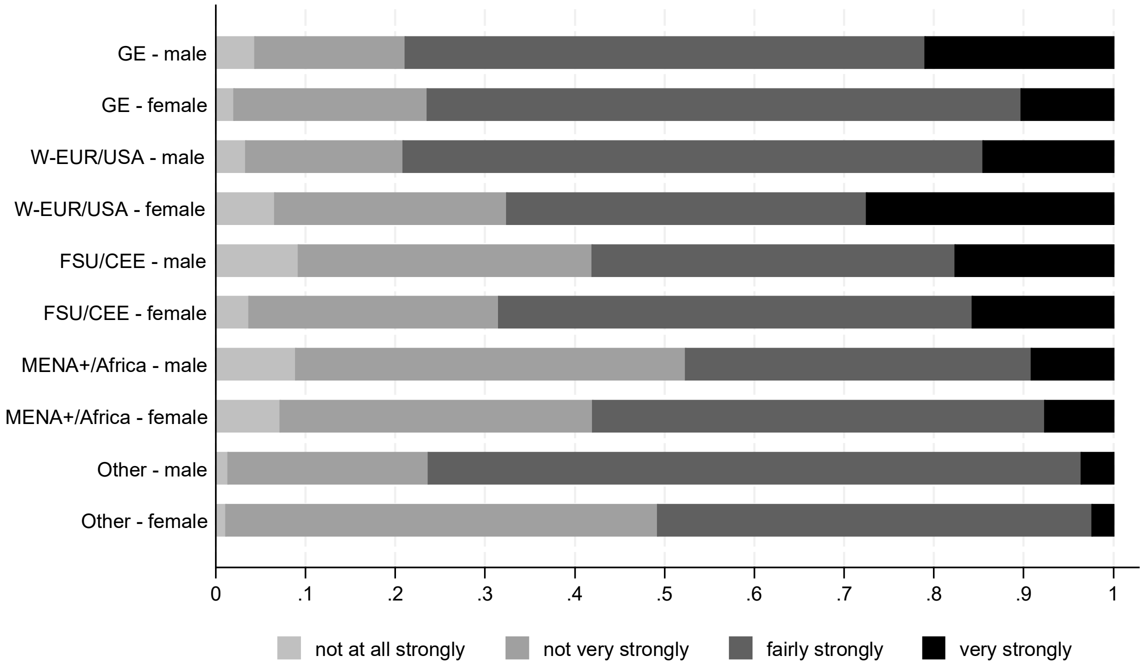


Source: CILS4EU, wave 5, wave 3 sample, weighted results.

## Appendix

Figure A1: Unfair treatment by police and security guards by origin groups, distribution of answers

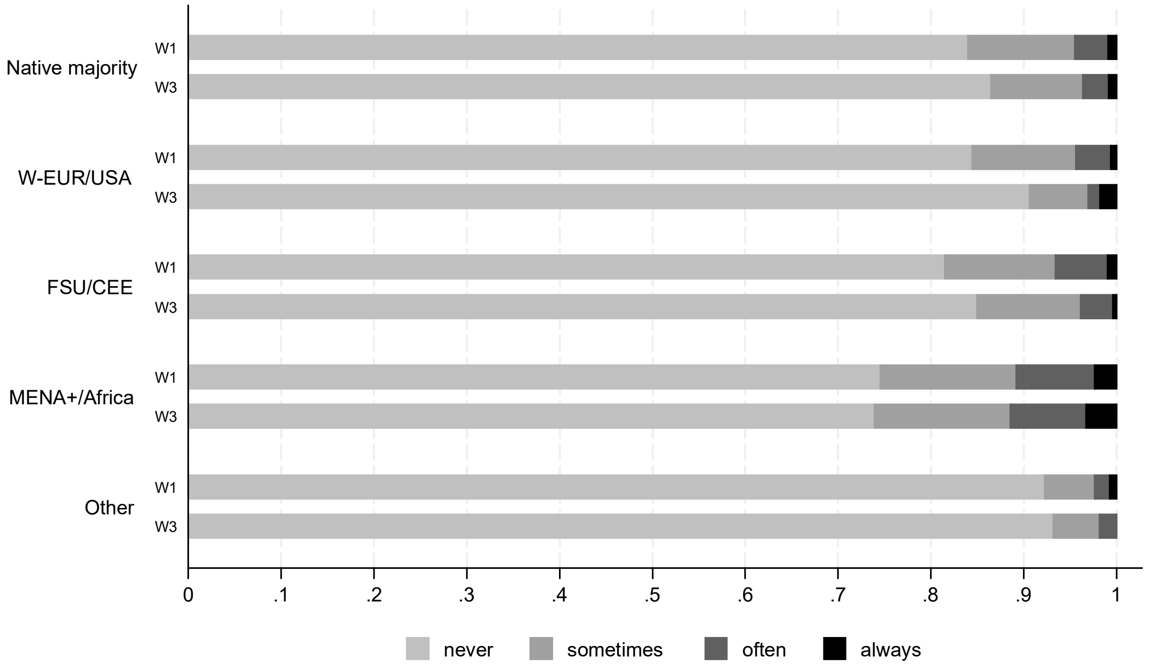


Source: CILS4EU, waves 1-3 (1.2.0, 2.3.0, 3.3.0), unbalanced sample, weighted results.
Notes: W1=Wave 1, W3=Wave 3.

Figure 2: Trust in police by origin groups, distribution of answers

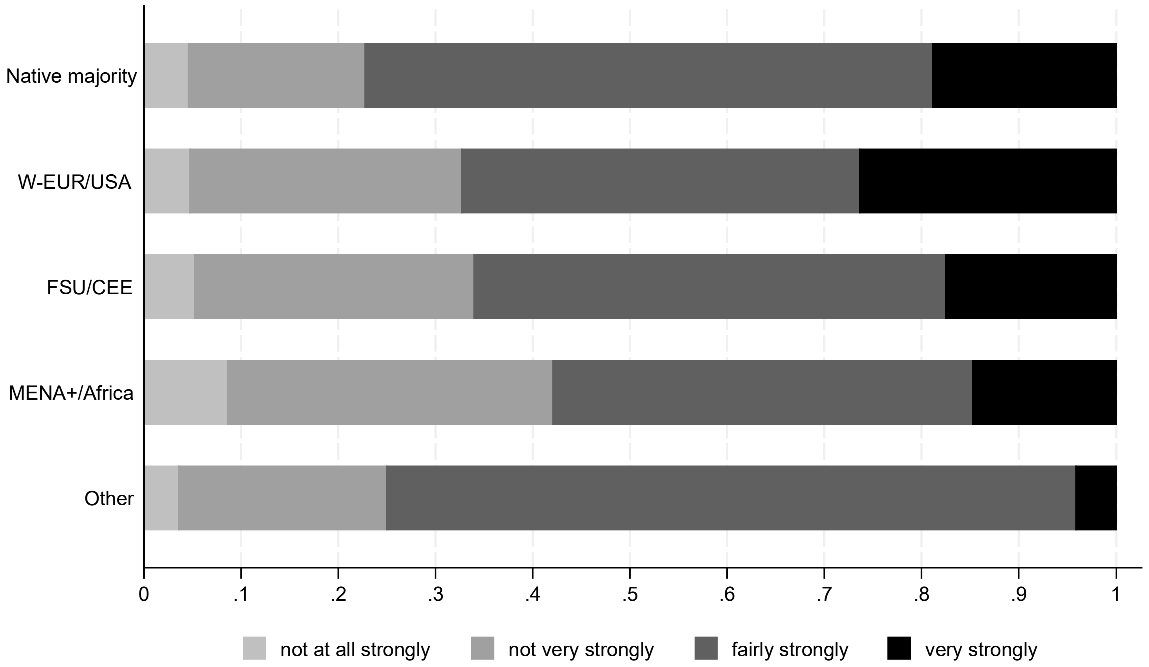


Source: CILS4EU, wave 5, wave 3 sample, weighted results.

Figure 4: Trust in courts by origin groups, distribution of answers

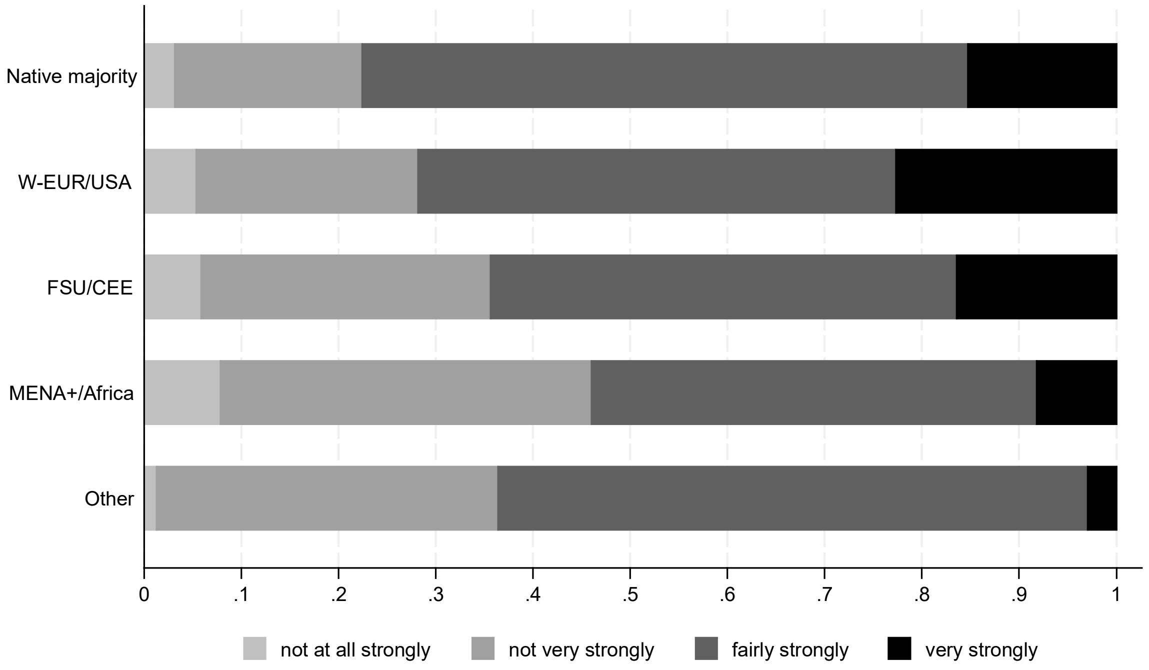


Source: CILS4EU, waves 5, wave 3 sample, weighted results.

Table A1: Full set of coefficients from OLS random effects regression with the dependent variable Feeling discriminated against by police/security guards

| | M1 | M2 | M3 |
|---|---|---|---|
| Origin groups (ref: native) | | | |
| W-EUR/USA | -0.05 | -0.02 | -0.01 |
| | (0.04) | (0.05) | (0.05) |
| x male | 0.05 | 0.04 | -0.02 |
| | (0.08) | (0.08) | (0.07) |
| FSU/CEE | -0.04 | -0.06 | -0.02 |
| | (0.03) | (0.04) | (0.04) |
| x male | $0.14^{*}$ | $0.14^{*}$ | $0.10^{+}$ |
| | (0.06) | (0.06) | (0.06) |
| MENA+/Africa | $-0.07^{*}$ | $-0.13^{*}$ | $-0.09^{+}$ |
| | (0.03) | (0.06) | (0.05) |
| x male | $0.34^{***}$ | $0.34^{***}$ | $0.29^{***}$ |
| | (0.06) | (0.06) | (0.06) |
| Other | $-0.08^{*}$ | $-0.10^{*}$ | -0.06 |
| | (0.04) | (0.05) | (0.05) |
| x male | 0.15 | $0.19^{+}$ | 0.15 |
| | (0.11) | (0.11) | (0.10) |
| Male | $0.08^{**}$ | $0.07^{**}$ | 0.03 |
| | (0.02) | (0.02) | (0.02) |
| Year of birth (ref.: 1995) | | | |
| Before 1994 | | $0.12^{**}$ | $0.07^{+}$ |
| | | (0.04) | (0.04) |
| After 1995 | | -0.03 | -0.02 |
| | | (0.02) | (0.02) |
| 1st generation immigrant | | 0.02 | 0.00 |
| | | (0.06) | (0.06) |
| Age at migration | | -0.01 | -0.00 |
| | | (0.01) | (0.01) |
| Religious denomination (ref.: no religion) | | | |
| Christian | | 0.01 | 0.02 |
| | | (0.03) | (0.03) |
| Muslim | | $0.13^{*}$ | $0.16^{*}$ |
| | | (0.06) | (0.06) |
| Other | | 0.03 | 0.05 |
| | | (0.09) | (0.08) |
| Importance of religion (ref.: not at all important) | | | |
| Not very important | | $-0.06^{*}$ | -0.02 |
| | | (0.02) | (0.02) |
| Fairly important | | $-0.08^{**}$ | -0.03 |
| | | (0.03) | (0.03) |
| Very important | | $-0.08^{*}$ | -0.02 |
| | | (0.04) | (0.03) |
| Parents' highest education (ref.: University) | | | |
| No degree/primary degree | | -0.04 | -0.03 |
| | | (0.05) | (0.05) |
| Secondary degree | | 0.02 | 0.02 |
| | | (0.03) | (0.03) |
| Unknown | | 0.06 | 0.09 |

| | | |
|---|---|---|
| | (0.15) | (0.14) |
| Parents' highest ISEI | 0.00 | 0.00 |
| | (0.00) | (0.00) |
| TV consumption in foreign language (ref.: no time at all) | | |
| Less than 1 hour a day | 0.12 | 0.13+ |
| | (0.08) | (0.07) |
| About 1 hour a day | -0.09 | -0.09 |
| | (0.06) | (0.06) |
| About 2 hours a day | -0.03 | -0.02 |
| | (0.06) | (0.05) |
| More than 2 hours a day | -0.11* | -0.11* |
| | (0.04) | (0.04) |
| Only German spoken at home | 0.03 | 0.04 |
| | (0.04) | (0.03) |
| Share of natives in neighborhood (ref.: almost all/all) | | |
| None or very few | 0.16* | 0.11 |
| | (0.08) | (0.07) |
| A few | 0.06 | 0.04 |
| | (0.04) | (0.04) |
| About half | 0.03 | 0.01 |
| | (0.03) | (0.03) |
| A lot | 0.03 | 0.03 |
| | (0.02) | (0.02) |
| Share of native friends (ref.: almost all/all) | | |
| None or very few | 0.16*** | 0.12** |
| | (0.05) | (0.04) |
| A few | 0.13*** | 0.10** |
| | (0.03) | (0.03) |
| About half | 0.11*** | 0.09** |
| | (0.03) | (0.03) |
| A lot | 0.06** | 0.04* |
| | (0.02) | (0.02) |
| Deliberately damaged others' things | | 0.17** |
| | | (0.05) |
| Stolen something from a shop/other people | | 0.01 |
| | | (0.07) |
| Carried a knife or weapon | | 0.28*** |
| | | (0.05) |
| Been very drunk | | 0.09*** |
| | | (0.03) |
| How often drink alcohol (ref.: never) | | |
| Weekly | | 0.05+ |
| | | (0.03) |
| Less often | | 0.04* |
| | | (0.02) |
| How often smoke cigarettes (ref.: never) | | |
| Weekly | | 0.14*** |
| | | (0.03) |
| Less often | | 0.04 |
| | | (0.03) |

| | | | |
|---|---|---|---|
| How often use drugs (ref.: never) | | | |
| Weekly | | | 0.43** |
| | | | (0.14) |
| Less often | | | 0.24*** |
| | | | (0.06) |
| | | | |
| Wave 3 | -0.03* | -0.05** | -0.04** |
| | (0.01) | (0.02) | (0.02) |
| Immigrant proportion in school W1 (ref.: 0-10%) | | | |
| 10-30% | 0.05* | 0.03 | 0.04* |
| | (0.02) | (0.02) | (0.02) |
| 30-60% | 0.08* | 0.03 | 0.05+ |
| | (0.03) | (0.03) | (0.03) |
| 60-100% | 0.13*** | 0.05 | 0.08* |
| | (0.04) | (0.04) | (0.04) |
| School type in W1 (ref.: Lower secondary school) | | | |
| School combining several tracks | -0.11 | -0.10 | -0.06 |
| | (0.07) | (0.07) | (0.07) |
| Intermediate secondary school | -0.06+ | -0.06+ | -0.01 |
| | (0.03) | (0.03) | (0.03) |
| Comprehensive school | -0.06 | -0.06 | -0.01 |
| | (0.04) | (0.04) | (0.04) |
| Upper secondary school | -0.11** | -0.11** | -0.05 |
| | (0.04) | (0.04) | (0.04) |
| School for special needs | 0.21 | 0.20 | 0.26+ |
| | (0.17) | (0.17) | (0.15) |
| Rudolf-Steiner school | -0.15** | -0.16** | -0.13* |
| | (0.05) | (0.05) | (0.06) |
| Intercept | -0.10 | -0.19 | -0.47** |
| | (0.09) | (0.12) | (0.17) |
| Observations | 4,185 | 4,185 | 4,185 |

Source: CILS4EU, waves 1-3 (1.2.0, 2.3.0, 3.3.0), unweighted. Unbalanced sample of respondents in wave 1 and wave 3 with information on either trust in police or trust in courts in wave 5. All models additionally control for federal state of school in Wave 1.

Note: + p < 0.1, * p < 0.05, ** p < 0.01, *** p < 0.001, panel-robust standard errors in parentheses.

Table A2: Feeling discriminated against by police/security guards, selected results from ordinal logistic regression (marginal effects from the random effect models)

| | M1 | M2 | M3 |
|---|---|---|---|
| Origin groups (ref: native) | | | |
| W-EUR/USA | -0.31 | -0.12 | 0.05 |
| | (0.41) | (0.42) | (0.42) |
| x male | 0.32 | 0.18 | -0.16 |
| | (0.56) | (0.55) | (0.52) |
| FSU/CEE | -0.32 | -0.25 | 0.01 |
| | (0.28) | (0.35) | (0.34) |
| x male | 0.84* | 0.78* | 0.58 |
| | (0.39) | (0.38) | (0.38) |
| MENA+/Africa | -0.64* | -0.77+ | -0.52 |
| | (0.28) | (0.39) | (0.39) |
| x male | 1.73*** | 1.74*** | 1.49*** |
| | (0.36) | (0.35) | (0.34) |
| Other | -0.68 | -0.69 | -0.42 |
| | (0.61) | (0.61) | (0.62) |
| x male | 1.06 | 1.33 | 1.03 |
| | (0.83) | (0.81) | (0.81) |
| Male | 0.55** | 0.50** | 0.28+ |
| | (0.17) | (0.17) | (0.16) |

Note: + p < 0.1, * p < 0.05, ** p < 0.01, *** p < 0.001; robust standard errors in parentheses, N=4,185. Unbalanced sample of respondents in wave 1 and wave 3 with information on either trust in police or trust in courts in wave 5, unweighted results.

Table A3: Feeling discriminated against by police/security guards, selected results from ordinal logistic regression (marginal effects from the random effect models)

| | M1 | M2 | M3 |
|---|---|---|---|
| Origin group (ref. native) | | | |
| W-EUR/USA | -0.04 | 0.02 | 0.14 |
| | (0.31) | (0.32) | (0.31) |
| x male | 0.33 | 0.21 | 0.09 |
| | (0.43) | (0.42) | (0.40) |
| FSU/CEE | -0.39+ | -0.49+ | -0.23 |
| | (0.23) | (0.29) | (0.28) |
| x male | 0.88** | 0.87** | 0.79** |
| | (0.30) | (0.30) | (0.29) |
| MENA+/Africa | -0.48* | -0.47 | -0.29 |
| | (0.21) | (0.30) | (0.28) |
| x male | 1.41*** | 1.45*** | 1.33*** |
| | (0.26) | (0.26) | (0.25) |
| Other | -0.77 | -1.00* | -0.85+ |
| | (0.49) | (0.49) | (0.49) |
| x male | 1.06+ | 1.40* | 1.49* |
| | (0.64) | (0.62) | (0.61) |
| Male | 0.66*** | 0.60*** | 0.28* |
| | (0.14) | (0.14) | (0.13) |

Note: + p < 0.1, * p < 0.05, ** p < 0.01, *** p < 0.001; robust standard errors in parentheses, N=6,246. Unbalanced sample of respondents in wave 1 and wave 3, unweighted results.

Table A4: Coefficients for selected origin groups from OLS random effects regression with the dependent variable Feeling discriminated against by police/security guards

| | M1 | M2 | M3 |
|---|---|---|---|
| Origin groups (ref: native) | | | |
| MENA+ | -0.07 | -0.16* | -0.13 |
| | (0.06) | (0.08) | (0.08) |
| x male | 0.34* | 0.34* | 0.33* |
| | (0.14) | (0.14) | (0.13) |
| Turkey | -0.08* | -0.17** | -0.14* |
| | (0.03) | (0.06) | (0.06) |
| x male | 0.36*** | 0.36*** | 0.28*** |
| | (0.07) | (0.07) | (0.07) |
| Other Africa | 0.06 | 0.07 | 0.07 |
| | (0.09) | (0.09) | (0.09) |
| x male | 0.12 | 0.13 | 0.21 |
| | (0.25) | (0.24) | (0.25) |
| Male | 0.08** | 0.07** | 0.03 |
| | (0.02) | (0.02) | (0.02) |

Note: + p < 0.1, * p < 0.05, ** p < 0.01, *** p < 0.001; robust standard errors in parentheses, N=4,185. Unbalanced sample of respondents in wave 1 and wave 3 with information on either trust in police or trust in courts in wave 5.

Table A5: Trust in police, selected results of the ordinal logistic regression with the dependent variable Feeling discriminated against by police/security guards (marginal effects)

| | M1 | M2 | M3 | M4 |
|---|---|---|---|---|
| Origin groups (ref: native) | | | | |
| W-EUR/USA | -0.12 | -0.01 | -0.04 | -0.09 |
| | (0.24) | (0.26) | (0.27) | (0.27) |
| x male | -0.30 | -0.21 | -0.08 | -0.08 |
| | (0.34) | (0.35) | (0.35) | (0.36) |
| FSU/CEE | -0.21 | -0.09 | -0.13 | -0.17 |
| | (0.16) | (0.20) | (0.21) | (0.21) |
| x male | 0.00 | -0.07 | -0.00 | 0.06 |
| | (0.26) | (0.27) | (0.27) | (0.27) |
| MENA+/Africa | -0.40** | -0.30 | -0.38 | -0.46+ |
| | (0.15) | (0.23) | (0.24) | (0.24) |
| x male | -0.43+ | -0.57* | -0.45+ | -0.17 |
| | (0.22) | (0.23) | (0.24) | (0.24) |
| Other | -0.58+ | -0.54+ | -0.58+ | -0.66+ |
| | (0.31) | (0.33) | (0.32) | (0.34) |
| x male | 0.34 | 0.01 | 0.10 | 0.22 |
| | (0.47) | (0.48) | (0.49) | (0.50) |
| Male | -0.18+ | -0.16 | -0.10 | -0.08 |
| | (0.10) | (0.10) | (0.11) | (0.11) |

Source: CILS4EU, waves 1-3 (1.2.0, 2.3.0, 3.3.0), and CILS4EU-DE, wave 5 (6.0.0), unweighted, including stratum. All models additionally control for federal state of school in Wave 1.
Note: + p < 0.1, * p < 0.05, ** p < 0.01, *** p < 0.001; robust standard errors in parentheses, N=2,386. For the variable "discrimination by police" a maximum value from waves 1 and 3 is taken.

Table A6: Coefficients for selected origin groups from OLS random effects regression with the dependent variable "Trust in police"

| | M1 | M2 | M3 | M4 |
|---|---|---|---|---|
| Origin groups (ref: native) | | | | |
| MENA+ | -0.14 | -0.12 | -0.13 | -0.17 |
| | (0.14) | (0.16) | (0.15) | (0.15) |
| x male | -0.04 | -0.09 | -0.08 | 0.01 |
| | (0.19) | (0.19) | (0.19) | (0.19) |
| Turkey | -0.14* | -0.09 | -0.12 | -0.16 |
| | (0.07) | (0.11) | (0.11) | (0.10) |
| x male | -0.26* | -0.29** | -0.23* | -0.11 |
| | (0.11) | (0.11) | (0.11) | (0.11) |
| Other Africa | -0.42* | -0.37* | -0.37* | -0.31+ |
| | (0.17) | (0.17) | (0.17) | (0.17) |
| x male | 0.16 | 0.00 | -0.02 | -0.03 |
| | (0.28) | (0.28) | (0.28) | (0.27) |
| Male | -0.10* | -0.09* | -0.06 | -0.05 |
| | (0.04) | (0.04) | (0.04) | (0.04) |

Source: CILS4EU, waves 1-3 (1.2.0, 2.3.0, 3.3.0), and CILS4EU-DE, wave 5 (6.0.0), unweighted, including stratum. All models additionally control for federal state of school in Wave 1.
Note: + p < 0.1, * p < 0.05, ** p < 0.01, *** p < 0.001; robust standard errors in parentheses, N=2,386. For the variable "discrimination by police" a maximum value from waves 1 and 3 is taken.

Table A7: Trust in police, full set of results from the OLS regression

| | M1 | M2 | M3 | M4 |
|---|---|---|---|---|
| Origin groups (ref: native) | | | | |
| W-EUR/USA | -0.06 | -0.04 | -0.05 | -0.07 |
| | (0.10) | (0.10) | (0.10) | (0.10) |
| x male | -0.12 | -0.06 | -0.01 | -0.01 |
| | (0.14) | (0.14) | (0.14) | (0.14) |
| FSU/CEE | -0.09 | -0.05 | -0.07 | -0.09 |
| | (0.07) | (0.08) | (0.08) | (0.08) |
| x male | 0.02 | -0.02 | 0.01 | 0.05 |
| | (0.11) | (0.11) | (0.11) | (0.10) |
| MENA+/Africa | -0.16** | -0.13 | -0.16+ | -0.19* |
| | (0.06) | (0.10) | (0.09) | (0.09) |
| x male | -0.18+ | -0.22* | -0.18+ | -0.07 |
| | (0.10) | (0.09) | (0.10) | (0.09) |
| Other | -0.25+ | -0.24+ | -0.25+ | -0.27* |
| | (0.13) | (0.14) | (0.14) | (0.13) |
| x male | 0.15 | -0.01 | 0.01 | 0.05 |
| | (0.21) | (0.20) | (0.20) | (0.20) |
| Male | -0.10* | -0.09* | -0.06 | -0.05 |
| | (0.04) | (0.04) | (0.04) | (0.04) |
| Year of birth (ref.: 1995) | | | | |
| Before 1995 | | -0.14* | -0.10 | -0.07 |
| | | (0.06) | (0.06) | (0.06) |
| After 1995 | | 0.04 | 0.04 | 0.03 |
| | | (0.03) | (0.03) | (0.03) |
| 1st generation immigrant | | -0.01 | 0.01 | 0.01 |
| | | (0.12) | (0.11) | (0.12) |
| Age at migration | | 0.04* | 0.04* | 0.03* |
| | | (0.02) | (0.02) | (0.02) |
| Religious denomination (ref.: no religion) | | | | |
| Christian | | -0.03 | -0.04 | -0.02 |
| | | (0.06) | (0.06) | (0.06) |
| Muslim | | 0.01 | -0.00 | 0.03 |
| | | (0.10) | (0.10) | (0.10) |
| Other | | 0.27+ | 0.28+ | 0.27+ |
| | | (0.16) | (0.15) | (0.15) |
| Importance of religion (ref.: not at all important) | | | | |
| Not very important | | 0.18*** | 0.14** | 0.12** |
| | | (0.05) | (0.05) | (0.05) |
| Fairly important | | 0.20*** | 0.16** | 0.15** |
| | | (0.05) | (0.05) | (0.05) |
| Very important | | 0.20** | 0.16* | 0.17* |
| | | (0.07) | (0.07) | (0.07) |
| Parents' highest education (ref.: University) | | | | |
| No degree/primary degree | | 0.09 | 0.07 | 0.05 |
| | | (0.08) | (0.08) | (0.08) |
| Secondary degree | | -0.08+ | -0.08+ | -0.08+ |
| | | (0.05) | (0.05) | (0.04) |
| Unknown | | -0.54* | -0.59* | -0.56* |

| | | | |
|---|---|---|---|
| | (0.25) | (0.27) | (0.25) |
| Parents' highest ISEI | -0.00 | 0.00 | 0.00 |
| | (0.00) | (0.00) | (0.00) |
| TV consumption in foreign language (ref.: no time at all) | | | |
| Less than 1 hour a day | -0.12 | -0.13 | -0.10 |
| | (0.16) | (0.15) | (0.15) |
| About 1 hour a day | -0.15 | -0.15 | -0.19 |
| | (0.17) | (0.17) | (0.17) |
| About 2 hours a day | 0.10 | 0.08 | 0.04 |
| | (0.08) | (0.08) | (0.08) |
| More than 2 hours a day | 0.00 | -0.01 | -0.04 |
| | (0.10) | (0.10) | (0.10) |
| No other language than German spoken at home | 0.05 | 0.05 | 0.05 |
| | (0.06) | (0.06) | (0.06) |
| Share of natives in neighborhood (ref.: almost all/all) | | | |
| None or very few | -0.24* | -0.23* | -0.18+ |
| | (0.11) | (0.11) | (0.11) |
| A few | -0.10 | -0.09 | -0.07 |
| | (0.07) | (0.07) | (0.07) |
| About half | -0.14* | -0.13* | -0.12* |
| | (0.06) | (0.06) | (0.06) |
| A lot | -0.11** | -0.11** | -0.09* |
| | (0.04) | (0.04) | (0.04) |
| Share of native friends (ref.: almost all/all) | | | |
| None or very few | -0.34*** | -0.29*** | -0.25** |
| | (0.08) | (0.08) | (0.08) |
| A few | -0.26*** | -0.21*** | -0.18** |
| | (0.07) | (0.06) | (0.06) |
| About half | -0.09 | -0.07 | -0.04 |
| | (0.05) | (0.05) | (0.05) |
| A lot | -0.06 | -0.05 | -0.04 |
| | (0.04) | (0.04) | (0.04) |
| Deliberately damaged others’ things | | -0.16 | -0.16 |
| | | (0.11) | (0.11) |
| Stolen something from a shop/ other people | | -0.14 | -0.09 |
| | | (0.12) | (0.12) |
| Carried a knife or weapon | | -0.12 | -0.05 |
| | | (0.08) | (0.08) |
| Been very drunk | | -0.03 | -0.01 |
| | | (0.04) | (0.04) |
| How often drink alcohol (ref.: never) | | | |
| Weekly | | 0.02 | 0.04 |
| | | (0.06) | (0.06) |
| Less often | | 0.00 | 0.01 |
| | | (0.05) | (0.05) |
| How often smoke cigarettes (ref.: never) | | | |
| Weekly | | -0.17*** | -0.10* |
| | | (0.05) | (0.05) |

| | | | | |
|---|---|---|---|---|
| Less often | | | -0.07 | -0.02 |
| | | | (0.06) | (0.06) |
| How often use drugs (ref.: never) | | | | |
| Weekly | | | -0.55*** | -0.44** |
| | | | (0.16) | (0.15) |
| Less often | | | -0.27** | -0.24** |
| | | | (0.09) | (0.09) |
| Feeling discriminated against by police/security guards (max value W1W3) | | | | |
| Sometimes | | | | -0.31*** |
| | | | | (0.05) |
| Often | | | | -0.43*** |
| | | | | (0.08) |
| Always | | | | -0.72*** |
| | | | | (0.13) |
| Immigrant proportion in school W1 (ref.: 0-10%) | | | | |
| 10-30% | -0.04 | -0.01 | -0.01 | 0.00 |
| | (0.05) | (0.05) | (0.05) | (0.05) |
| 30-60% | -0.11+ | -0.03 | -0.03 | -0.02 |
| | (0.06) | (0.06) | (0.06) | (0.06) |
| 60-100% | -0.09 | 0.02 | 0.01 | 0.04 |
| | (0.07) | (0.07) | (0.07) | (0.07) |
| School type in W1 (ref.: Lower secondary school) | | | | |
| School combining several tracks | 0.04 | -0.00 | -0.06 | -0.07 |
| | (0.17) | (0.18) | (0.17) | (0.17) |
| Intermediate secondary school | 0.03 | 0.02 | -0.02 | -0.03 |
| | (0.05) | (0.05) | (0.05) | (0.05) |
| Comprehensive school | 0.03 | 0.03 | -0.01 | -0.01 |
| | (0.06) | (0.06) | (0.06) | (0.06) |
| Upper secondary school | 0.19** | 0.17* | 0.11+ | 0.09 |
| | (0.06) | (0.06) | (0.07) | (0.06) |
| School for special needs | 0.22 | 0.24 | 0.19 | 0.20 |
| | (0.19) | (0.19) | (0.19) | (0.19) |
| Rudolf-Steiner school | -0.23 | -0.25 | -0.22 | -0.29* |
| | (0.15) | (0.16) | (0.15) | (0.15) |
| Intercept | 2.02*** | 2.01*** | 2.09*** | 2.08*** |
| | (0.08) | (0.14) | (0.14) | (0.14) |
| Observations | 2,386 | 2,386 | 2,386 | 2,386 |

Source: CILS4EU, waves 1-3 (1.2.0, 2.3.0, 3.3.0) and CILS4EU-DE, wave 5 (6.0.0), unweighted. All models additionally control for federal state of school in Wave 1.

Note: + $p < 0.1$, * $p < 0.05$, ** $p < 0.01$, *** $p < 0.001$; robust standard errors in parentheses. For the variable “discrimination by police” a maximum value from waves 1 and 3 is taken.

Table A8: Trust in courts, selected results of the ordinal logistic regression (marginal effects)

| | M1 | M2 | M3 | M4 |
|---|---|---|---|---|
| Origin groups (ref: native) | | | | |
| W-EUR/USA | 0.07 | 0.23 | 0.20 | 0.19 |
| | (0.24) | (0.27) | (0.27) | (0.27) |
| x male | -0.37 | -0.28 | -0.16 | -0.17 |
| | (0.33) | (0.34) | (0.34) | (0.34) |
| FSU/CEE | -0.02 | 0.24 | 0.22 | 0.19 |
| | (0.16) | (0.21) | (0.21) | (0.21) |
| x male | -0.51$^{+}$ | -0.59$^{*}$ | -0.51$^{+}$ | -0.46$^{+}$ |
| | (0.27) | (0.27) | (0.27) | (0.27) |
| MENA+/Africa | -0.31$^{*}$ | -0.03 | -0.08 | -0.12 |
| | (0.16) | (0.23) | (0.23) | (0.23) |
| x male | -0.58$^{*}$ | -0.67$^{**}$ | -0.59$^{*}$ | -0.42$^{+}$ |
| | (0.23) | (0.24) | (0.25) | (0.25) |
| Other | -0.59$^{+}$ | -0.47 | -0.48 | -0.52 |
| | (0.31) | (0.31) | (0.32) | (0.33) |
| x male | 0.27 | 0.03 | 0.08 | 0.14 |
| | (0.46) | (0.45) | (0.45) | (0.46) |
| Male | 0.28$^{**}$ | 0.31$^{**}$ | 0.33$^{**}$ | 0.36$^{**}$ |
| | (0.10) | (0.10) | (0.11) | (0.11) |

Source: CILS4EU, waves 1-3 (1.2.0, 2.3.0, 3.3.0), and CILS4EU-DE, wave 5 (6.0.0), unweighted, including stratum. All models additionally control for federal state of school in Wave 1.
Note: + p < 0.1, * p < 0.05, ** p < 0.01, *** p < 0.001; robust standard errors in parentheses, N=2,363. For the variable "discrimination by police" a maximum value from waves 1 and 3 is taken.

Table A9: Coefficients for selected origin groups from OLS random effects regression with the dependent variable "Trust in courts"

| | M1 | M2 | M3 | M4 |
|---|---|---|---|---|
| Origin groups (ref: native) | | | | |
| MENA+ | -0.18 | -0.08 | -0.08 | -0.10 |
| | (0.14) | (0.15) | (0.15) | (0.15) |
| x male | 0.07 | 0.03 | 0.02 | 0.08 |
| | (0.20) | (0.20) | (0.19) | (0.19) |
| Turkey | -0.09 | 0.03 | 0.02 | -0.01 |
| | (0.07) | (0.10) | (0.10) | (0.10) |
| x male | -0.31$^{**}$ | -0.33$^{**}$ | -0.30$^{**}$ | -0.23$^{*}$ |
| | (0.10) | (0.10) | (0.11) | (0.11) |
| Other Africa | -0.25 | -0.21 | -0.21 | -0.17 |
| | (0.18) | (0.19) | (0.18) | (0.18) |
| x male | 0.07 | -0.01 | -0.03 | -0.04 |
| | (0.30) | (0.31) | (0.30) | (0.31) |
| Male | 0.09$^{*}$ | 0.10$^{**}$ | 0.11$^{**}$ | 0.12$^{**}$ |
| | (0.04) | (0.04) | (0.04) | (0.04) |

Source: CILS4EU, waves 1-3 (1.2.0, 2.3.0, 3.3.0), and CILS4EU-DE, wave 5 (6.0.0), unweighted, including stratum. All models additionally control for federal state of school in Wave 1.
Note: + p < 0.1, * p < 0.05, ** p < 0.01, *** p < 0.001; robust standard errors in parentheses, N=2,363. For the variable "discrimination by police" a maximum value from waves 1 and 3 is taken.

Table A 10: Trust in courts, full set of results of the OLS regression

| | M1 | M2 | M3 | M4 |
|---|---|---|---|---|
| Origin groups (ref: native) | | | | |
| W-EUR/USA | 0.01 | 0.06 | 0.04 | 0.03 |
| | (0.09) | (0.10) | (0.10) | (0.10) |
| x male | -0.13 | -0.10 | -0.06 | -0.06 |
| | (0.13) | (0.13) | (0.13) | (0.13) |
| FSU/CEE | -0.01 | 0.07 | 0.05 | 0.05 |
| | (0.06) | (0.08) | (0.08) | (0.08) |
| x male | -0.18$^{+}$ | -0.21$^{*}$ | -0.18$^{+}$ | -0.16 |
| | (0.10) | (0.10) | (0.10) | (0.10) |
| MENA+/Africa | -0.12$^{+}$ | -0.02 | -0.03 | -0.05 |
| | (0.06) | (0.09) | (0.09) | (0.09) |
| x male | -0.19$^{*}$ | -0.23$^{*}$ | -0.20$^{*}$ | -0.14 |
| | (0.09) | (0.09) | (0.09) | (0.09) |
| Other | -0.21$^{+}$ | -0.18 | -0.18 | -0.19 |
| | (0.12) | (0.12) | (0.12) | (0.12) |
| x male | 0.12 | 0.00 | 0.02 | 0.04 |
| | (0.18) | (0.17) | (0.17) | (0.17) |
| Male | 0.09$^{*}$ | 0.10$^{**}$ | 0.11$^{**}$ | 0.12$^{**}$ |
| | (0.04) | (0.04) | (0.04) | (0.04) |
| Year of birth (ref.: 1995) | | | | |
| Before 1995 | | -0.13$^{*}$ | -0.11$^{+}$ | -0.09 |
| | | (0.06) | (0.06) | (0.05) |
| After 1995 | | 0.02 | 0.01 | 0.01 |
| | | (0.03) | (0.03) | (0.03) |
| 1st generation immigrant | | -0.02 | -0.00 | 0.00 |
| | | (0.10) | (0.10) | (0.10) |
| Age at migration | | 0.02 | 0.02 | 0.02 |
| | | (0.01) | (0.01) | (0.01) |
| Religious denomination (ref.: no religion) | | | | |
| Christian | | -0.11$^{*}$ | -0.12$^{*}$ | -0.11$^{*}$ |
| | | (0.05) | (0.05) | (0.05) |
| Muslim | | -0.15$^{+}$ | -0.14 | -0.12 |
| | | (0.09) | (0.09) | (0.09) |
| Other | | 0.12 | 0.12 | 0.11 |
| | | (0.15) | (0.15) | (0.15) |
| Importance of religion (ref.: not at all important) | | | | |
| Not very important | | 0.16$^{***}$ | 0.13$^{**}$ | 0.12$^{**}$ |
| | | (0.04) | (0.04) | (0.04) |
| Fairly important | | 0.19$^{***}$ | 0.17$^{**}$ | 0.16$^{**}$ |
| | | (0.05) | (0.05) | (0.05) |
| Very important | | 0.19$^{**}$ | 0.17$^{**}$ | 0.18$^{**}$ |
| | | (0.06) | (0.07) | (0.07) |
| Parents' highest education (ref.: University) | | | | |
| No degree/primary degree | | 0.08 | 0.06 | 0.04 |
| | | (0.08) | (0.08) | (0.08) |
| Secondary degree | | -0.10$^{*}$ | -0.10$^{*}$ | -0.10$^{*}$ |
| | | (0.04) | (0.04) | (0.04) |
| Unknown | | -0.70$^{**}$ | -0.74$^{**}$ | -0.73$^{**}$ |
| | | (0.27) | (0.28) | (0.27) |

| | | | |
|---|---|---|---|
| Parents' highest ISEI | -0.00 | 0.00 | 0.00 |
| | (0.00) | (0.00) | (0.00) |
| TV consumption in foreign language (ref.: no time at all) | | | |
| Less than 1 hour a day | -0.09 | -0.07 | -0.05 |
| | (0.17) | (0.16) | (0.16) |
| About 1 hour a day | -0.02 | -0.03 | -0.05 |
| | (0.15) | (0.14) | (0.15) |
| About 2 hours a day | 0.08 | 0.07 | 0.05 |
| | (0.09) | (0.09) | (0.09) |
| More than 2 hours a day | 0.00 | 0.01 | -0.01 |
| | (0.10) | (0.10) | (0.10) |
| No other language than German spoken at home | 0.07 | 0.07 | 0.08 |
| | (0.05) | (0.05) | (0.05) |
| Share of natives in neighborhood (ref.: almost all/all) | | | |
| None or very few | -0.25* | -0.24* | -0.21+ |
| | (0.11) | (0.11) | (0.11) |
| A few | -0.17** | -0.16** | -0.15* |
| | (0.06) | (0.06) | (0.06) |
| About half | -0.13* | -0.12* | -0.11* |
| | (0.06) | (0.06) | (0.06) |
| A lot | -0.06+ | -0.06 | -0.05 |
| | (0.04) | (0.04) | (0.04) |
| Share of native friends (ref.: almost all/all) | | | |
| None or very few | -0.22** | -0.19* | -0.16* |
| | (0.08) | (0.08) | (0.08) |
| A few | -0.10+ | -0.08 | -0.05 |
| | (0.06) | (0.06) | (0.06) |
| About half | -0.07 | -0.07 | -0.05 |
| | (0.05) | (0.05) | (0.05) |
| A lot | 0.03 | 0.03 | 0.04 |
| | (0.04) | (0.04) | (0.04) |
| Deliberately damaged others' things | | -0.27* | -0.26* |
| | | (0.11) | (0.11) |
| Stolen something from a shop/ other people | | -0.08 | -0.05 |
| | | (0.12) | (0.12) |
| Carried a knife or weapon | | -0.07 | -0.03 |
| | | (0.07) | (0.07) |
| Been very drunk | | -0.02 | -0.01 |
| | | (0.04) | (0.04) |
| How often drink alcohol (ref.: never) | | | |
| Weekly | | 0.06 | 0.08 |
| | | (0.06) | (0.06) |
| Less often | | 0.04 | 0.05 |
| | | (0.05) | (0.05) |
| How often smoke cigarettes (ref.: never) | | | |
| Weekly | | -0.14** | -0.09* |
| | | (0.05) | (0.05) |
| Less often | | -0.05 | -0.02 |
| | | (0.05) | (0.05) |

| | | | | |
|---|---|---|---|---|
| How often use drugs (ref.: never) | | | | |
| Weekly | | | -0.08 | -0.00 |
| | | | (0.14) | (0.14) |
| Less often | | | -0.14$^{+}$ | -0.12 |
| | | | (0.08) | (0.08) |
| Feeling discriminated against by police/security guards (max value W1W3) | | | | |
| Sometimes | | | | -0.18$^{***}$ |
| | | | | (0.04) |
| Often | | | | -0.27$^{***}$ |
| | | | | (0.07) |
| Always | | | | -0.46$^{***}$ |
| | | | | (0.12) |
| Immigrant proportion in school W1 (ref.: 0-10%) | | | | |
| 10-30% | -0.03 | -0.01 | -0.02 | -0.01 |
| | (0.04) | (0.04) | (0.04) | (0.05) |
| 30-60% | -0.10$^{+}$ | -0.04 | -0.04 | -0.03 |
| | (0.06) | (0.06) | (0.06) | (0.06) |
| 60-100% | -0.09 | 0.03 | 0.03 | 0.05 |
| | (0.06) | (0.07) | (0.07) | (0.07) |
| School type in W1 (ref.: Lower secondary school) | | | | |
| School combining several tracks | 0.03 | 0.00 | -0.04 | -0.05 |
| | (0.15) | (0.16) | (0.15) | (0.15) |
| Intermediate secondary school | 0.09$^{+}$ | 0.10$^{+}$ | 0.07 | 0.06 |
| | (0.05) | (0.05) | (0.05) | (0.05) |
| Comprehensive school | 0.10$^{+}$ | 0.10$^{+}$ | 0.07 | 0.07 |
| | (0.06) | (0.06) | (0.06) | (0.06) |
| Upper secondary school | 0.30$^{***}$ | 0.27$^{***}$ | 0.24$^{***}$ | 0.22$^{***}$ |
| | (0.06) | (0.06) | (0.06) | (0.06) |
| School for special needs | -0.06 | -0.03 | -0.06 | -0.05 |
| | (0.17) | (0.18) | (0.18) | (0.18) |
| Rudolf-Steiner school | -0.07 | -0.08 | -0.06 | -0.10 |
| | (0.15) | (0.16) | (0.16) | (0.16) |
| Intercept | 1.72$^{***}$ | 1.75$^{***}$ | 1.78$^{***}$ | 1.78$^{***}$ |
| | (0.08) | (0.13) | (0.14) | (0.14) |
| Observations | 2,363 | 2,363 | 2,363 | 2,363 |

Source: CILS4EU, waves 1-3 (1.2.0, 2.3.0, 3.3.0) and CILS4EU-DE, wave 5 (6.0.0), unweighted. All models additionally control for federal state of school in Wave 1.

Note: + $p < 0.1$, * $p < 0.05$, ** $p < 0.01$, *** $p < 0.001$; robust standard errors in parentheses.
